# MST1 promotes microglial pyroptosis and neuroinflammation in alzheimer’s disease by regulating the novel DPP8/NLRP1/Caspase-1/GSDMD-N axis

**DOI:** 10.1186/s12974-026-03732-3

**Published:** 2026-02-13

**Authors:** Dongqing Cui, Shuangwu Liu, Yanxia Liu, Shuqi Luo, Yurui Sheng, Shuaiyong Zhang, Pengfei Lin

**Affiliations:** 1 Department of Neurology, Research Institute of Neuromuscular and Neurodegenerative Diseases, Shandong Provincial Key Laboratory of Mitochondrial Medicine and Rare Diseases, Qilu Hospital of Shandong University, Jinan, Shandong 250012 China; 2 School of Nursing and Rehabilitation, Cheeloo College of Medicine, Shandong University, Jinan, 250012 China; 3 Department of Neurology, The Second Hospital of Shandong University, Shandong University, Jinan, 250033 China; 4 Department of Radiology, The First Affiliated Hospital of Shandong First Medical University & Shandong Provincial Qianfoshan Hospital, Jinan, 250000 China; 5 Department of Clinical Medicine, The Second Hospital of Shandong University, Shandong University, Jinan, 250033 China

**Keywords:** Alzheimer's disease, DPP8, MST1, Microglia, Neuroinflammation, Pyroptosis

## Abstract

Alzheimer’s disease (AD) is a progressive neurodegenerative disorder characterized by β-amyloid (Aβ) induced disruption of brain homeostasis, leading to neuronal damage and cognitive impairment. Increasing evidence confirms that microglia-driven neuroinflammation serves as a core mechanism driving the progression of AD. Mammalian Ste20-like kinase 1 (MST1) plays a crucial regulatory role in apoptosis, immune inflammation, and oxidative stress. Our team’s previous research revealed that MST1 regulates mitochondrial oxidative stress in neurons, contributing to the pathogenesis of AD. Here, we show that MST1 is activated as p-MST1 in the peripheral blood of AD patients, the serum of 5xFAD mice, and the hippocampal and cortical brain tissues of 5xFAD mice, an effect which was associated with microglial pyroptosis under chronic inflammatory stimulation. Knocking down MST1 in hippocampal and cortical tissues of 5xFAD mice improved cognitive deficits, reduced p-tau protein levels, and alleviated neurodegeneration and neuroinflammatory responses. Concurrently, MST1 knockdown suppressed abnormal microglial activation, decreased inflammatory cytokine release, and ultimately mitigated microglial pyroptosis. Mechanistically, we found that MST1 knockdown modulated DPP8 protein expression, thereby regulating the NLRP1/Caspase-1/GSDMD-N signaling axis to inhibit microglial pyroptosis and attenuate neuroimmune inflammation. In summary, MST1 knockdown improved AD disease progression by preventing disruption to the immune-inflammatory homeostasis of microglia. Therefore, we propose targeting MST1 as a promising therapeutic strategy to halt neuroinflammation and progression in Alzheimer’s disease.

**Graphical Abstract:**

**Figure:**
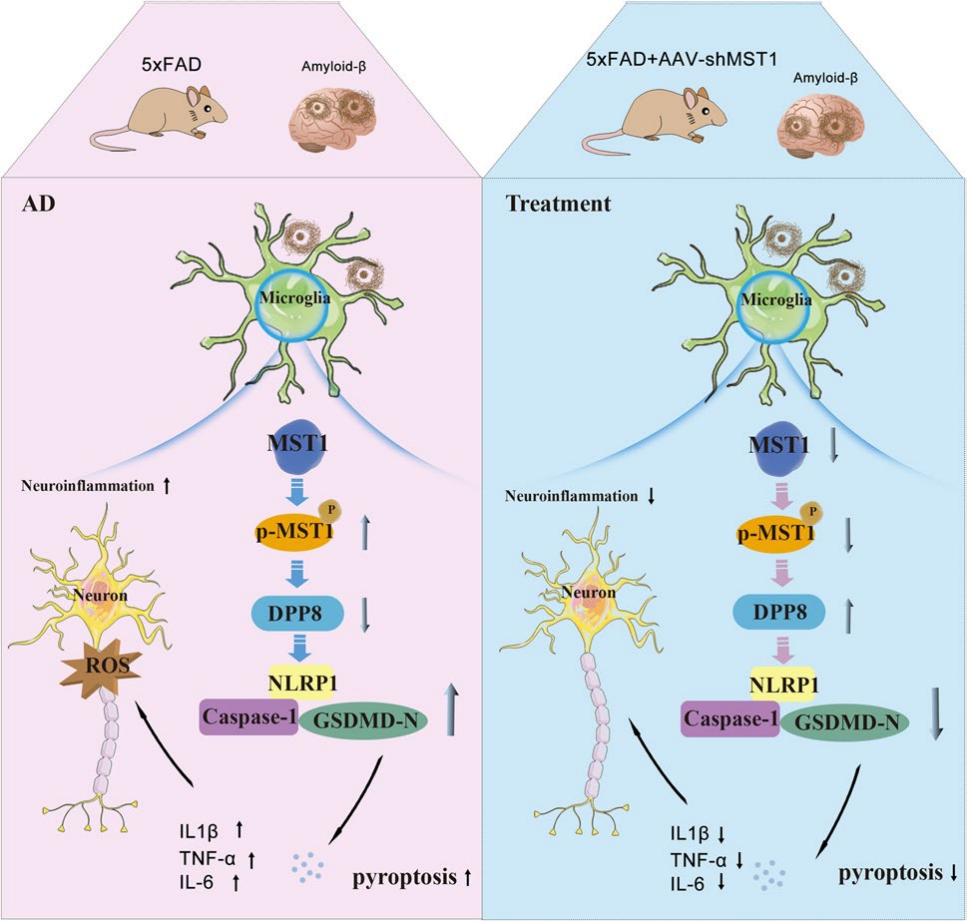
Schematic diagram illustrating the mechanism by which MST1-mediated regulation of DPP8 drives microglial pyroptosis via the NLRP1/Caspase-1/GSDMD-N axis in Alzheimer's disease.

**Supplementary Information:**

The online version contains supplementary material available at 10.1186/s12974-026-03732-3.

## Introduction

Alzheimer’s disease (AD) is a progressive neurodegenerative disorder characterized by β-amyloid plaque deposition, neurofibrillary tangles composed of hyperphosphorylated tau protein, and neuroinflammation [1]. While significant advances have been made in characterizing these features, the precise pathogenesis of AD remains incompletely understood, and effective disease-modifying therapies are still lacking [2, 3]. Consequently, there is an urgent need to elucidate the underlying pathophysiological mechanisms and identify novel diagnostic biomarkers and therapeutic targets.

Accumulating evidence from genetic and molecular studies highlights the central role of neuroimmune dysregulation in AD pathogenesis [4–6]. Microglia, the resident immune cells of the central nervous system, are essential for maintaining brain homeostasis [7]. In AD, however, microglia undergo pathological activation and contribute to disease progression through multiple mechanisms, including the release of inflammatory cytokines, synaptic remodeling, phagocytic activity, and dynamic phenotypic switching between the pro-inflammatory M1 and anti-inflammatory M2 states [8–10]. Notably, microglial activation is intimately associated with pyroptosis, a highly inflammatory form of programmed cell death [11]. This process is typically mediated by inflammasome complexes such as NLRP3/1, which activate caspase-1, leading to gasdermin D cleavage and subsequent release of pro-inflammatory cytokines including IL-1β and IL-18 [12–14]. Pyroptosis has been increasingly implicated in AD pathology, where it may perpetuate a vicious cycle of inflammatory signaling and cellular damage, thereby accelerating neurodegenerative processes [15–17].

The Hippo signaling pathway, an evolutionarily conserved regulator of cell proliferation, apoptosis, and immune homeostasis, has recently been linked to various pathological conditions, including neurodegenerative and neuroinflammatory disorders [18–21]. Mammalian Ste20-like kinase 1 (MST1), a core upstream component of the Hippo pathway, has been demonstrated to influence synaptic plasticity, mitochondrial function, and neuronal survival in experimental models of AD [22, 23]. Beyond its neuronal functions, MST1 is increasingly recognized as a critical modulator of neuroimmune responses [24, 25]. For instance, recent studies have shown that maintains NLRP3 inflammasome homeostasis by mediating feedback loops and has been identified as a potential therapeutic target for regulating inflammatory processes [26]. Additional research focusing on the central nervous system reveals that MST1 influences immune inflammatory responses following cerebral ischemia-reperfusion injury by modulating the activation state of microglia, suggesting its broader involvement in neuroinflammatory cascades [27]. Nevertheless, the specific functional contribution of MST1 to microglial pyroptosis within the context of AD pathology remains largely unexplored.

Given the lack of exploration regarding MST1’s role in microglial pyroptosis within AD, proactive mechanism investigation is essential. DPP8 has recently garnered significant academic attention. As a serine protease structurally homologous to the DPP4 subfamily, DPP8 is characterized by its regulation of cellular metabolism and cellular immune inflammation [28]. Notably, recent studies have reported that DPP8 inhibitors act as universal activators of pyroptosis-associated inflammasome NLRP1 alleles, thereby activating pro-caspase-1 and inducing pyroptosis in immune cells [29–32]. For instance, DPP8 inhibitors exert therapeutic effects on acute myeloid leukemia by inducing pyroptosis [33]; DPP8 inhibition induces pro-caspase-1-dependent pyroptosis in monocytes and macrophages [34]. However, research on DPP8 in AD has been limited to the discovery of the DPP8 locus on chromosome 15 as a novel gene associated with memory decline [35]. Nevertheless, the relationship between MST1 and DPP8 remains unresolved. We firmly believe that unraveling the enigmatic connection between MST1, DPP8, and microglial pyroptosis in AD will significantly contribute to identifying novel therapeutic targets for the disease.

In this study, we aim to systematically investigate the role of MST1 in AD pathogenesis, with particular emphasis on its regulatory function in microglial pyroptosis and neuroinflammation. First, we assess the potential of phosphorylated MST1 (p-MST1) in peripheral blood as a diagnostic biomarker for AD using clinical baseline data. Second, we employ an AD mouse model to determine whether MST1 modulates neuronal injury and cognitive impairment through the regulation of microglial pyroptosis. Finally, using in vitro AD models, we elucidate the molecular mechanisms by which MST1 governs microglial inflammatory responses. Our findings reveal a novel role for MST1 in AD progression and provide a rationale for targeting the MST1-pyroptosis axis as a potential therapeutic strategy for Alzheimer’s disease.

## Materials and methods

### Study participants

The experimental design and implementation of this study strictly adhered to the ethical requirements of the Declaration of Helsinki. Patients with AD and gender- and age-matched healthy controls were recruited from the Qilu Hospital of Shandong University, and all participants included in the study signed an informed consent form. It was approved by the Research Ethics Committee of Qilu Hospital of Shandong University (Approval No. DWLL-2021-264). The information collected in the study included demographic data (age, gender) and general clinical data (The demographic and clinical parameters of the cohort are detailed in Table S1). The Mini-Mental State Examination (MMSE) and Montreal Cognitive Assessment (MoCA) were used to evaluate the global cognitive function of enrolled AD patients, and their peripheral blood samples were collected. The diagnosis of AD was made in strict accordance with the diagnostic criteria developed by the National Institute on Aging-Alzheimer’s Association (NIA-AA) in 2018 [27].

### Mice

Six-month-old male 5xFAD and C57BL/6 J mice were utilized in this study, both obtained from Beijing Huafukang Biotechnology Co., Ltd. All animal experiments strictly adhered to the Animal Experimentation Ethics Guidelines of the National Institutes of Health (NIH, USA). On the basis of ensuring the scientific validity and reproducibility of the experiment, the number of animals used was reduced through optimized experimental design, and measures were taken to minimize their stress and suffering. The experimental mice were housed in a specific pathogen-free (SPF) barrier environment, with the following housing conditions: dry and well-ventilated environment, temperature controlled at 24 ± 2 °C, light cycle of 12 h per day, and free access to food and water. All animal experimental procedures and studies were approved by the Ethics Committee of the Qilu Hospital of Shandong University. The experimental animals were randomly assigned to the respective groups after genotype identification to ensure comparable baseline conditions among groups. Subsequently, behavioral testing, tissue processing, immunofluorescence imaging, as well as quantitative analysis of images/bands were all conducted independently by investigators who were blinded to the group allocation.

### Cell culture and treatment

Both BV2 cells (a microglial cell line) and HT22 cells (a hippocampal neuronal cell line) were purchased from the Shanghai Cell Bank of the Chinese Academy of Sciences (Shanghai, China). Both cell lines were cultured in Dulbecco’s Modified Eagle Medium (DMEM, Gibco, California, USA, 11995065) supplemented with 10% fetal bovine serum (FBS, Lonsera, S7100-001 S) and 1% penicillin-streptomycin mixture (Solarbio, Beijing, China, P1410), under standard cell culture conditions of 37 °C and 5% CO_2_ [36].

Amyloid-β (1–42) oligomers were purchased from Shanghai Qiangyao Biotechnology Co., Ltd (Cat. No: 04010064589). A working solution was prepared by first dissolving 1 mg of Amyloid-β (1–42) oligomers powder in 20µL of sterile dimethyl sulfoxide (DMSO), followed by the addition of 220µL of sterile phosphate-buffered saline (PBS). The mixture was then sonicated to facilitate complete dissolution and thoroughly mixed. BV2 cells were treated with different concentrations of Aβ_1–42_ oligomers (5, 10, 20, and 40µM) for 24 h to establish the cellular model and to examine the changes in MST1 and p-MST1 expression (The final concentration of DMSO is less than 0.5%). For all control group cells, culture medium containing the corresponding concentration of DMSO alone was added as a vehicle control to exclude any potential interference from the solvent.

Lipopolysaccharide (LPS) powder (Sigma, USA, L2880) was dissolved in sterile PBS to prepare a working solution with a concentration of 1 µg/ml. Adenosine triphosphate (ATP) powder (MedChemExpress, USA, HY-B2176) was dissolved in sterile PBS to prepare a working solution with a concentration of 5mM. BV2 cells were first treated with 1 µg/mL LPS for 23 h, followed by stimulation with 5mM ATP to induce pyroptosis [37].

### Cell transfection

Both small interfering RNAs (siRNAs), namely siMST1 and siDPP8, were purchased from Shandong Keyanyun Biotechnology Co., Ltd. The target sequences are 5′-GCUGGUUCUGUAUCUGAUATT-3′ for siMST1 and 5′-GGACAGACAGAGACUUAUATT-3′ for siDPP8. For the experiment, BV2 cells were seeded into 6-well culture plates at a density of 1 × 10^5^cells per well. Once the cells had grown to 70–90% confluency, they were transfected using Lipofectamine™ 3000 Transfection Reagent (L3000-015, Invitrogen), following the manufacturer’s operating instructions.

### Intracerebroventricular injections

The AAV-shRNA-MST1 carrying the Iba1-specific promoter was purchased from Hanheng Company. Its sequence is as follows: 5′-GCCAGATTGTTGCAATCAAGC-3′. Our previous study has confirmed that this sequence can significantly downregulates MST1 expression [21]. Following the method described previously [38, 39], the mice were anaesthetised with 2% isoflurane and fixed to a stereotaxic instrument. Subsequently, 2µL of AAV was slowly injected into each lateral ventricle using a microinjector at a rate of 0.2µL/min. The injection stereotaxic coordinates (relative to Bregma) were: AP − 0.6 mm, ML ± 1.2 mm, DV − 2.2 mm. Once the injection was complete, the needle was left in place for 10 min before the wound was sutured and the mice received postoperative care. Behavioral assessments were conducted one month after the injection, following which the mice were euthanized and their brains were harvested for subsequent experiments.

### Morris water maze test

One month after AAV injection, the Morris water maze test was conducted to evaluate the spatial learning and cognitive functions of mice in each group [21]. The detailed experimental protocol is as follows: The instrument used in the experiment was a large circular water tank (1.5 m in diameter, 35 cm in height), with reference markers placed on four sides of the inner wall of the tank. The water temperature in the tank was maintained at 20–22 °C, and a circular platform was placed in the target quadrant. On day 0 of the experiment, the mice underwent adaptive training, followed by 5 consecutive days of formal training. Each day, 4 training sessions were carried out starting from different quadrants (each training session lasted 60 s, with a 1-hour interval between two sessions); after finding the platform, each mouse was allowed to stay on it for 20 s. On the final day of training (i.e., day 5), the platform was removed, and the time that the mice spent in the target quadrant within 60 s, as well as the number of times they crossed the original platform position were measured. All experimental data were automatically collected and recorded by a computer video tracking system (SMART software, Panlab, Spain). A single animal was used as the statistical unit. Behavioral parameters of mice in each group were analyzed using GraphPad Prism 8.0 software, including swimming speed, escape latency, number of platform crossings, and total time spent in the target quadrant. The behavioral testing, video tracking, and data analysis were all performed by experimenters who were blinded to the group assignments. In addition, mice that exhibited persistent floating, failure to swim actively, or inability to complete the training procedures were excluded from the analysis.

### Western blotting analysis

Protein samples from cells and brain tissues were extracted using RIPA lysis buffer (Beyotime, Shanghai, China, P0013B) supplemented with protease and phosphatase inhibitors. The protein concentration was then measured using a BCA assay kit (Solaibio, Beijing, China, PC0020). 5×Loading Buffer was added to the protein samples, which were then heated at 95 °C for 10 min. Equal amounts of protein the samples (30–60 µg) were separated using 8–12% SDS-PAGE gels and transferred to PVDF membranes (Millipore, USA, ISEQ00010). The membranes were blocked with a 5% non-fat milk solution for 1.5 h, then incubated with primary antibodies at 4 °C overnight, and then with secondary antibodies at room temperature for 1 h. Detailed information of all antibodies used is provided in Table S2. Protein expression was detected using enhanced chemiluminescence (ECL) Western blotting detection reagents (Merck Millipore, USA, WBKLS0050). Images were captured with a Tanon4800 chemiluminescence imager (Shanghai, China). Quantitative analysis of the protein images was performed using Image J software. The target protein was normalized to the internal control protein GAPDH or β-actin.

### Immunofluorescence (IF) staining

After anesthesia, mice were subjected to cardiac perfusion for brain tissue collection. The brain tissues were immersed in 4% paraformaldehyde for 24-hour fixation, followed by gradient dehydration with sucrose solution. Subsequently, the brain tissues were embedded in OCT, and then cut into 20 μm thick sections using a cryostat. The tissue sections were immersed in 1× antigen retrieval solution (Beyotime, Shanghai, P0083) at room temperature for 5 min, followed by three washes with PBS for 5 min each. The sections were then incubated in a permeabilization and blocking solution consisting of 0.2% Triton X-100 and 5% goat serum, and kept at room temperature for 1 h. The diluted primary antibodies were added dropwise to the tissue sections and incubated overnight at 4 °C. The primary antibodies used were: anti-p-MST1(Thr183) antibody (1:100), anti-iNOS antibody (1:100), anti-Iba1 antibody (1:100), anti-GFAP antibody (1:100), anti-p-tau(S396) antibody (1:100), anti-DPP8 antibody (1:100), and anti-GSDMD-N antibody (1:100). The next day, the sections were removed and washed with PBS three times for 5 min each. Fluorescent secondary antibodies (goat anti-mouse/rabbit IgG Alexa Fluor Plus 488, 1:500, Abcam; goat anti-rabbit IgG Alexa Fluor 647, 1:500, Abcam) were added dropwise, and the sections were incubated at room temperature for 1 h in the dark. After washing with PBS again, mounting medium containing DAPI was added dropwise to the sections and coverslips were placed on top. Images of the hippocampal DG region and the cerebral cortex were acquired using a laser scanning confocal microscope (Zeiss LSM 800, Germany). Representative regions were visualized and imaged using 20×, 40×, and 100× oil-immersion objectives. The fluorescence intensity in the hippocampus and cortex of 3–4 animals per group was analyzed and quantified using ImageJ software. Further details can be found in the Supplementary Methods.

Cells adjusted to an appropriate concentration were seeded in confocal dishes, or used for cell climbing slices. After completion of the intervention treatment, the cells were rinsed with PBS, followed by fixation with cell immunofixative. A permeabilization and blocking solution containing 0.2% Triton X-100 and 5% goat serum was added to the confocal dishes and incubated with the cells at room temperature for 1 h, followed by three washes with PBS for 5 min each. The diluted primary antibody was then applied to fully immerse the cells and incubated overnight at 4 °C. The primary antibodies used included: anti-p-MST1(Thr183) antibody (1:100), anti-Iba1 antibody (1:100), anti-NLRP1 antibody (1:100), anti-GSDMD-N antibody (1:100), anti-IL-1β antibody (1:100), and anti-NeuN antibody (1:100). After overnight incubation, the confocal dishes were washed three times with PBS for 5 min each. Fluorescent secondary antibodies were then applied (goat anti-rabbit IgG Alexa Fluor Plus 488, 1:500, Abcam; goat anti-rabbit IgG Alexa Fluor 647, 1:500, Abcam), and the dishes were incubated at room temperature for 1 h in the dark. After another series of PBS washes, an anti-fluorescence quenching mounting medium containing DAPI was added. Detailed information of all antibodies used is provided in Table S3.

To minimize potential bias introduced by the same individual during data processing, we employed a blinded design in which image acquisition and data analysis were performed by different researchers, thereby ensuring the objectivity and reliability of the results.

### Real-time quantitative polymerase chain reaction (RT-qPCR)

Total RNA was extracted from cells and brain tissues using the RNA-Quick Purification Kit (ES Science Biotech, Shanghai, RN002), following the manufacturer’s instructions. Absorbance was measured using a spectrophotometer to quantitatively analyse the RNA. Total RNA was reverse transcribed into cDNA using the Evo M-MLV Reverse Transcription Kit (Accurate Biotechnology Co., Ltd., AG11711) according to the manufacturer’s instructions. The reverse transcription reaction was performed under the following conditions: 37 °C for 15 min, followed by 85 °C for 5 s to terminate the reaction, and then held at 4 °C. Real-time quantitative PCR was conducted using SYBR qPCR Master Mix (Vazyme, Nanjing, China, Q711-02) on a QuantStudio 5 Real-Time PCR System (ThermoFisher™ Scientific, USA) to assess the mRNA expression levels of target genes. The amplification program was as follows: Step 1: 95 °C for 30 s (1 cycle); Step 2: 95 °C for 5 s and 60 °C for 30 s (40 cycles); Step 3: Dissociation stage (melting curve analysis) to verify amplification specificity. Gene-specific primers were used for all qPCR reactions. All relative gene expression analyses were conducted using the 2^(−ΔΔCt)^ method. The primer sequences used are provided in Table S4.

### Hematoxylin-eosin staining (HE)

The mouse brain tissues were paraffin-embedded and cut into 5 μm-thick sections. After dewaxing and rehydration, the sections were stained using a Hematoxylin-Eosin (HE) staining kit (Servicebio, Wuhan, China, G1076). Following the manufacturer’s instructions, the sections underwent hematoxylin staining, differentiation, bluing, and eosin staining, then dehydration and clearing. Finally, the sections were mounted with neutral balsam and covered with coverslips, and images were acquired by scanning with a digital slide scanner (NanoZoomer S60, Japan). Images of brain tissue from each group were examined to evaluate neuronal injury in the hippocampal subregions (DG, CA1, and CA3) and the cerebral cortex.

### Nissl staining

After paraffin embedding of mouse brain tissues, 5 μm-thick sections were cut. Following dewaxing and rehydration, the sections were stained using a Nissl staining kit (Servicebio, Wuhan, China, G1036) with the following steps: the sections were immersed in toluidine blue solution and stained at room temperature for 2–5 min; after rinsing with water, they were differentiated with 0.1% glacial acetic acid, and the degree of differentiation was controlled by timely observation under a microscope. After differentiation, the sections were placed in an oven for drying, and subsequent clearing and mounting processes were performed. Images were acquired using a digital pathology scanner. Subsequently, brain images from three mice in each group were used to assess neuronal alterations in the hippocampal subregions (DG, CA1, and CA3) as well as in the cerebral cortex. For each image, three randomly selected fields were quantitatively analyzed to evaluate the extent of neuronal injury. ImageJ software was used to count the number of dead and viable cells in the images, and the ratio between them was calculated [21].

### Cell viability assays

BV2 cells were seeded in 96-well plates at a density of 7000–8000 cells per well. After plasmid transfection and drug stimulation treatment, 10µL of CCK8 reagent (MedChemExpress, USA, HY-K0301) was added to each well, followed by incubation at 37 °C in the dark for 1–2 h. The optical density (OD) value at 450 nm was measured using a microplate reader (Biotek, Winooski, Vermont, USA).

### Lactate dehydrogenase (LDH) release assay

According to the reagent manufacturer’s instructions, the experiment was performed using the LDH kit (Nanjing Jiancheng Technology Co., Ltd, Nanjing, China, A020-2). Cell culture medium was collected and centrifuged at 1000 rpm at 4 °C for 5 min. The supernatant was taken for LDH detection, and the OD value at 450 nm was measured using a microplate reader (Biotek, Winooski, Vermont, USA). The level of LDH released from cells was calculated using the following formula: LDH Activity (U/L) = (*A*_determined_*−A*_control_*) / (A*_standard_*−A*_blank_*) × C*_standard_ *× N ×* 1000. where *N* represents the dilution factor of the sample prior to measurement, *C*_standard_ is the concentration of the standard solution (0.2µmol/mL), and 1000 is the conversion factor from mL to L. Finally, data from each experimental group were normalized to the control group to obtain the percentage of LDH release.

### Transwell measurement

To simulate the in vivo interaction between microglia and neuronal cells, we performed a BV2-HT22 co-culture experiment. The co-culture was conducted using a Transwell system with polycarbonate membranes (pore size: 0.4 μm). HT22 cells were seeded into the bottom chamber at a density of 1 × 10^5^ cells/well, while BV2 cells transfected with siRNA were seeded into the upper chamber at a density of 1 × 10^5^ cells/insert. The BV2 cells were first treated with LPS (1 µg/ml) for 23 h, followed by treatment with ATP (5 mM) for 1 h. Subsequently, the Transwell insert (containing the treated BV2 cells) was transferred into the wells housing HT22 cells, and then the two cell types were co-cultured for 24 h. During this co-culture period, soluble factors secreted by cells could be exchanged through the polycarbonate membrane inserts.

### Enzyme-linked immunosorbent assay (ELISA)

Cerebrospinal fluid samples were collected from mice, and p-MST1 (Thr183) levels were measured using a mouse ELISA kit according to the manufacturer’s instructions. Peripheral blood samples from humans and mice were centrifuged at 3500 rpm at 4 °C for 15 min. The upper serum layer was carefully separated, and the levels of p-MST1(Thr183) in the serum were measured using human-specific and mouse-specific ELISA kits, respectively, according to the manufacturers’ instructions. Additionally, the level of p-Tau181 in human serum was detected using a human p-Tau181 ELISA kit. Absorbance was determined at 450 nm using a microplate absorbance reader (Biotek, Vermont, USA).

After 24 h of co-culturing BV2 and HT22 cells, the cell culture medium was collected and centrifuged at 1000 rpm at 4 °C for 5 min. The concentrations of IL-6, IL-1β, and TNF-α in the cell culture supernatant were measured using an ELISA kit according to the manufacturer’s instructions. Absorbance was determined at 450 nm using a microplate absorbance reader (Biotek, Vermont, USA). Further details can be found in the Supplementary Methods.

### Cell death assessment by flow cytometry

To evaluate the damage to HT22 cells induced by pro-inflammatory cytokines secreted by BV2 cells, the Annexin V-FITC/PI Apoptosis Detection Kit (Vazyme, Nanjing, China, A211-01) was used to assess the apoptosis level of HT22 cells according to the manufacturer’s recommendations. After 24 h of co-culture of BV2 and HT22 cells, HT22 cells in the lower chamber were collected, washed twice with pre-cooled PBS, and resuspended in buffer. The cells were then incubated with Annexin V-FITC and PI for 10 min at room temperature in the dark, and the apoptosis level of HT22 cells was detected by flow cytometry. Early apoptotic and late apoptotic cell ratios were presented as the percentages of Annexin V⁺/PI⁻ and Annexin V⁺/PI⁺ labelled cells, respectively. The apoptotic rate of HT22 cells was the sum of the proportions of early apoptotic cells and late apoptotic cells. Data analysis was performed using FlowJo v10 software.

### TMT-based proteomic analysis

The identification and analysis of the TMT-liquid chromatography (LC)-tandem mass spectrometry (MS/MS) experiment were performed by Novogene (Beijing, China). The specific procedure is as follows: First, total proteins were extracted from mouse hippocampal tissues. After determining the protein concentration using the Bradford protein quantification kit, the proteins were labeled with TMT^®^ Mass Tagging Kits and Reagents (purchased from Thermo). Subsequently, the TMT-labeled samples mentioned above were subjected to liquid chromatography elution using the EASY-nLCTM 1200 nano UHPLC system. Then, mass spectrometry detection was carried out with the Q Exactive™ HF-X mass spectrometer. Further details can be found in the Supplementary Methods.

### Statistical analysis

Statistical analysis in this study was performed using GraphPad Prism 8.0 software. Quantitative data were expressed as mean ± standard error of the mean (SEM), while categorical variables were presented as frequency (%). The normality of the data was assessed via the Shapiro-Wilk test; the correlation between p-MST1 and other indicators was analyzed using Spearman correlation analysis; Multivariate logistic regression analysis was used to analyze the independent effects of each variable on the risk of Alzheimer’s disease. unpaired two-tailed Student’s t-test was employed to compare differences between two groups; and one-way analysis of variance (ANOVA) was used for comparing differences among multiple groups, followed by Tukey’s post-hoc multiple comparison analysis or Bonferroni multiple comparison test as needed. Proteomics data were analyzed using R software (Version 4.1.1). In all statistical analyses, a single animal or an independent biological sample was used as the unit of analysis. All experiments were independently repeated at least three times, and a *p*-value < 0.05 was considered statistically significant.

## Results

### The activation level of MST1 in the serum of AD patients is increased and correlates with disease severity

**Fig. 1:**
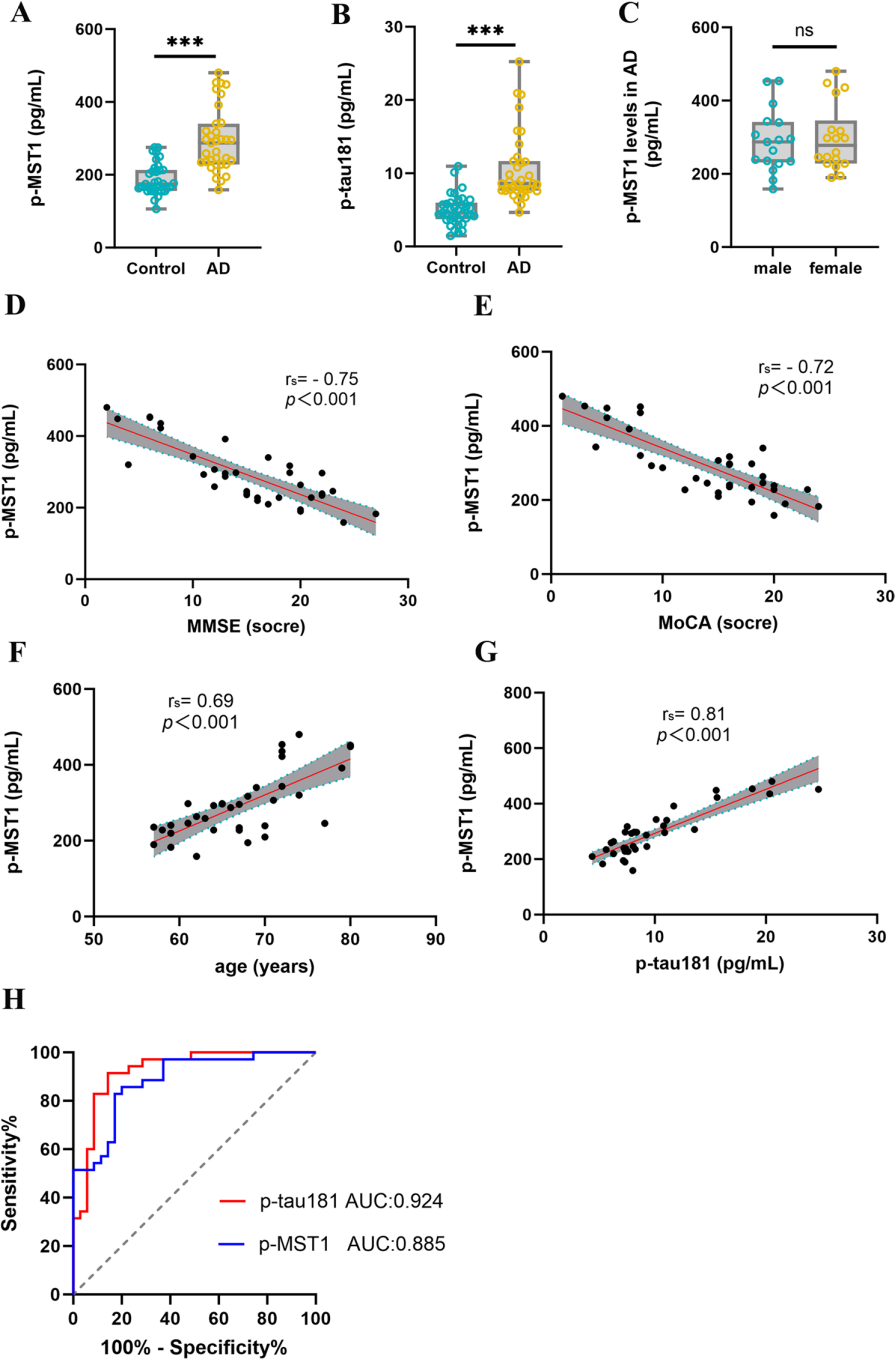
Analysis of the correlation between p-MST1 levels in serum and general clinical data in AD patients. Clinical data and serum samples were collected from 35 AD patients and 35 healthy controls (matched by gender and age) for protein detection and correlation analysis. **A** Scatter plot of serum p-MST1 expression levels in AD patients and healthy controls. **B** Scatter plot of serum p-tau181 expression levels in AD patients and healthy controls. **C** Scatter plot of p-MST1 expression in different gender groups of AD patients. **D** Correlation analysis between serum p-MST1 expression levels and MMSE scores in patients with AD. **E** Correlation analysis between serum p-MST1 expression levels and MoCA scores in patients with AD. **F** Correlation analysis between serum p-MST1 expression levels and age in patients with AD. **G** Correlation analysis between serum p-MST1 expression levels and serum p-tau181 levels in patients with AD. **H** ROC curve of p-MST1 and p-tau181 in AD. (The AUC value of the ROC curve represents the overall performance of a biomarker. An AUC of 0.5 indicates no difference from random chance, while an AUC of 1.0 indicates a biomarker with 100% specificity and sensitivity.) Two-tailed unpaired Student’s t-tests were used to evaluate statistical significance. Spearman correlation analysis was used to analyze the correlation between p-MST1 and clinical characteristics. Data are expressed as mean ± SEM; ***p < 0.001; *ns*, no significance; r_s_, correlation coefficient

MST1 is a kinase with multiple biological functions that can be activated by various stressful conditions. It is mainly activated through phosphorylation, transforming into p-MST1 and participating in the regulation of multiple biological functions. To evaluate the correlation between MST1 activation and clinical data in Alzheimer’s disease patients, as well as its potential diagnostic value, this study collected data from 35 age- and sex-matched patients with AD and 35 healthy control individuals. The demographic and clinical parameters of the cohort are detailed in Table S1. First, the levels of p-MST1 and p-tau181 were detected in the peripheral blood of the participants. Compared with the control group, the results showed that the levels of p-MST1 (Fig. 1A, *p* < 0.001) and p-tau181 (Fig. 1B, *p* < 0.001) in the serum of AD patients were significantly higher. However, there was no statistically significant difference in the p-MST1 level between male and female AD patients (Fig. 1C, *p* > 0.05). In this study, we employed a logistic regression model to analyze the effects of multiple variables on AD. The results of the logistic regression analysis indicated that there were no significant associations between age and sex with AD outcomes (*p* > 0.05), suggesting that the matching process effectively balanced the baseline characteristics between the two groups. Therefore, it can be inferred that the differences in age and sex between the control group and the AD group were no longer significant and were not potential confounding factors affecting the analyses. Furthermore, the logistic regression analysis revealed that p-MST1 (OR = 1.035, 95% CI [1.011–1.059], *p* = 0.004 < 0.05) and p-tau181 (OR = 2.471, 95% CI [1.428–4.275], *p* = 0.001 < 0.05) were independent risk factors for the occurrence of AD (Table S5).

To preliminarily clarify the correlation between MST1 activation and AD progression, we conducted Spearman correlation analysis on the levels of p-MST1 in the peripheral blood of AD patients with MMSE scores, MoCA scores, patients’ ages, and serum p-tau181 levels, respectively. The results showed that the levels of p-MST1 in the peripheral blood of AD patients were significantly negatively correlated with MMSE scores (Fig. 1D; r_s_ = − 0.75, *p* < 0.001) and also significantly negatively correlated with MoCA scores (Fig. 1E; r_s_ = − 0.72, *p* < 0.001). In contrast, this indicator was significantly positively correlated with patients’ ages (Fig. 1F; *r* = 0.69, *p* < 0.001) and also significantly positively correlated with serum p-Tau181 levels (Fig. 1G; *r* = 0.81, *p* < 0.001). The results of the present study indicate a correlation between the levels of p-MST1 in peripheral blood and the progression of AD. The Receiver Operating Characteristic Curve (ROC) was utilised to evaluate the diagnostic efficacy of p-MST1 and p-tau181 for AD. The results showed that the area under the ROC curve (AUC) of p-MST1 was 0.885, while the AUC of p-Tau181 was 0.924 (Fig. 1H). The findings of this study segment suggest that p-MST1 has the potential to serve as a promising candidate biomarker requiring validation for AD. The combination of these results leads to the conclusion that, in AD, MST1 is activated into p-MST1, which is associated with the severity of the disease and has the potential to serve as a diagnostic biomarker.

### MST1 serves as a risk gene involved in the pathogenesis of AD

**Fig. 2:**
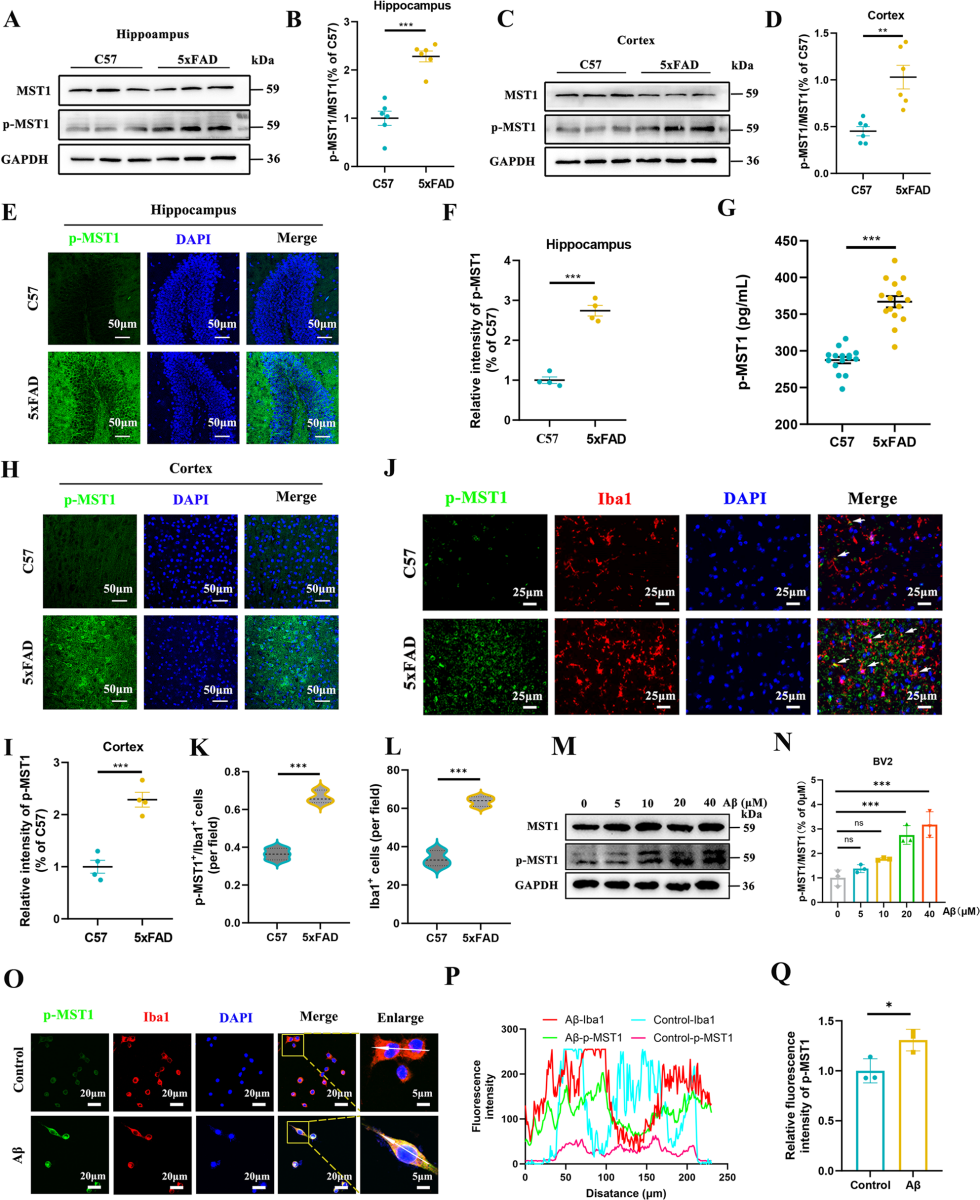
MST1 is activated in AD and can be expressed in microglia. **A**–**B**. Western blot analysis and quantification of total MST1 and p-MST1(Thr183) expression in hippocampal tissues of 6-month-old C57 and 5xFAD mice (*n* = 6 mice per group). **C**–**D**. Western blot analysis and quantification of total MST1 and p-MST1(Thr183) expression in cortex tissues of 6-month-old C57 and 5xFAD mice (*n* = 6 mice per group). **E**–**F**. Immunofluorescence images and quantitative analysis of p-MST1(Thr183) in the hippocampus of 6-month-old C57 and 5xFAD mice (*n* = 4 mice; 2 sections/mouse; 3 fields/section; data averaged per mouse, original magnification: × 20, scale bars is 50 μm). **G**. Scatter plot of serum p-MST1 levels in C57 and 5xFAD mice (*n* = 15 mice per group). **H**–**I**. Immunofluorescence images and quantitative analysis of p-MST1(Thr183) in the cortex of 6-month-old C57 and 5xFAD mice (*n* = 4 mice; 2 sections/mouse; 3 fields/section; data averaged per mouse, scale bar is 50 μm). **J**–**L**. p-MST1(Thr183) and Iba1 immunofluorescence co-localization images and co-localization degree analysis in 6-month-old C57 and 5xFAD mice, marked microglia with the Iba1 antibody (*n* = 3 mice; 2 sections/mouse; 3 fields/section; original magnification: × 20, scale bars is 50 μm). **M**. Western blot images of MST1 and p-MST1(Thr183) in BV2 cells treated with different concentrations of Aβ_1−42_. **N**. Quantitative analysis of p-MST1(Thr183) /MST1 (*n* = 3 mice per group). **O**. Confocal images analysis of p-MST1(Thr183) and Iba1 immunofluorescence staining in BV2 cells from the control group and the Aβ_1−42_ treated group, microglia were marked with the Iba1 antibody (scale bar is 10 μm, scale bar for enlarged insets is 5 μm). **P**. Fluorescence intensity of p-MST1(Thr183) and Iba1 in cells indicated by the white arrows. **Q**. Relative immunofluorescence intensity of p-MST1(Thr183) protein in different groups. Two-tailed unpaired Student’s t-tests and one-way ANOVA with Tukey’s multiple comparison test were used to evaluate statistical significance. Data are expressed as mean ± SEM, **p < 0.01, ***p < 0.001, *ns*, no significance

To investigate the expression and activation status of MST1 in AD, the 5xFAD mouse model and the Aβ_1−42_ induced BV2 cell model were employed. Firstly, the expression levels of MST1 and p-MST1 were detected quantitatively in the hippocampus and cortex of 6-month-old 5xFAD mice and wild-type (WT) mice. The activation level of MST1 was evaluated by calculating the p-MST1/MST1 ratio. The results of the western blot analysis demonstrated that the p-MST1/MST1 ratio in the hippocampus (Fig. 2A, B) and cortex (Fig. 2C, D) of 5xFAD mice was significantly higher than that observed in the WT control group. Immunofluorescence further confirmed that p-MST1 expression was significantly higher in the hippocampus (Fig. 2E, F) and cortex (Fig. 2H, I) of 5xFAD mice than in controls, consistent with the results of the Western blot analysis. Similarly, ELISA results showed that p-MST1 levels in both the serum (Fig. 2G) and cerebrospinal fluid (Fig. S1A) of 5xFAD mice were significantly elevated compared with those of the control group. Interestingly, RNA-seq results from our previous study suggested that MST1 could regulate neuroimmune inflammatory responses in 5xFAD mice [21]. Therefore, to evaluate the expression of p-MST1 in microglia, immunofluorescence co-staining experiments were performed, with microglia being labelled using an Iba1 antibody. The results showed that compared with the WT control group, the expression level of p-MST1 in microglia of 5xFAD mice was significantly elevated (Fig. 2J, K, L).

Furthermore, we verified the expression of p-MST1 in an in vitro BV2 cell model induced by Aβ_1−42_. Western blot results showed that, as the concentration of Aβ_1−42_ increased, MST1 activation into p-MST1 gradually increased, as evidenced by a concentration-dependent rise in the p-MST1/MST1 ratio (Fig. 2M, N). Immunofluorescence results also confirmed that p-MST1 was highly expressed in BV2 cells induced by Aβ_1−42_ (Fig. 2O, P, Q). Taken together, these findings suggest that p-MST1 is a key risk factor in the development of AD.

### Knockdown of MST1 improves cognitive function in 5xFAD mice

**Fig. 3:**
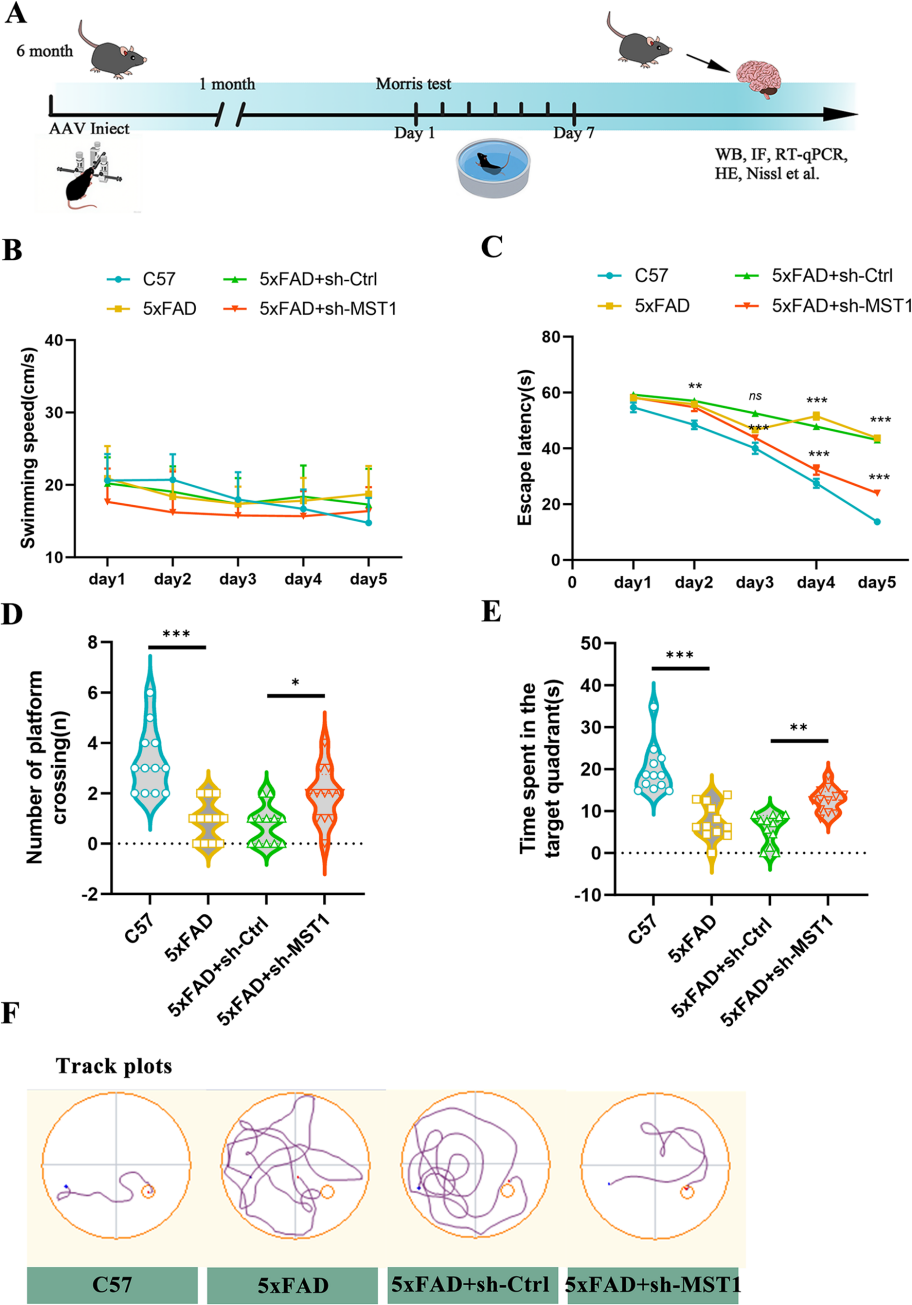
Knockdown of MST1 improves cognitive function in 5xFAD mice. **A** Experimental schedule diagram for stereotaxic injection into the lateral ventricles and related experiments. **B**–**F**. MWM test results of four groups of mice, including swimming speed (**B**), escape latency (**C**), number of platform crossings (**D**), total time spent in the target quadrant (**E**), representative traces on the fifth day of the MWM training period (**F**). (*n* = 12 mice per group). Two-way ANOVA with the Bonferroni multiple comparison test and One-way ANOVA with Tukey’s multiple comparison test were employed. Data are expressed as mean ± SEM, *p < 0.05, **p < 0.01, ***p < 0.001, *ns*, no significance

To further evaluate the critical role of MST1 in AD, we constructed AAV vectors carrying a microglia-specific promoter to mediate MST1 knockout, as well as corresponding empty vector AAV controls. The mice were divided into four groups: C57 group, 5xFAD group, 5xFAD + sh-Ctrl group, and 5xFAD + sh-MST1 group. The viruses were then injected into the lateral ventricles of mice to assess their effects on 6-month-old 5xFAD mice (experimental flowchart: Fig. 3A). One month after viral injection, we found that the virus carrying a microglia-specific promoter successfully mediated GFP expression in microglial cells (Fig. S1B, C), and the efficiency of MST1 knockout in the mouse hippocampus and cortex was validated at both the protein and gene levels. Western blot results showed that compared with the 5xFAD + sh-Ctrl group, MST1 protein expression in the hippocampus (Fig. S1D, E) and cortex (Fig. S1D, F) of the 5xFAD + sh-MST1 group was significantly lower. RT-qPCR results revealed a significant reduction in MST1 mRNA in the hippocampus (Fig. S1G) and cortex (Fig. S1H) of mice in the 5xFAD + sh-MST1 group. In addition, Western blot analysis showed that p-MST1 (Thr183) levels were significantly elevated in the hippocampal tissue of 5xFAD mice, indicating enhanced MST1 activation, whereas this activation was markedly attenuated following MST1 knockdown (Fig. S1I, J). These results indicated that MST1 was effectively inhibited in the hippocampus and cortex of 5xFAD mice.

One month after stereotaxic intracerebral virus injection, the Morris Water Maze Test was used to evaluate their spatial learning and memory abilities. The mice underwent five consecutive days of training, during which their swimming speed and escape latency were recorded. During this training phase, no significant differences in swimming speed were observed between the groups, indicating that MST1 knockdown does not impact the motor function of 5xFAD mice (Fig. 3B). Compared with the control group, 6-month-old 5xFAD mice exhibited a significantly longer escape latencies. However, 5xFAD mice with MST1 knockdown had markedly shorter escape latencies than those injected with an empty virus (Fig. 3C). Test phase results indicated that 5xFAD mice had significantly fewer platform crossings and spent less time in the target quadrant than the control group (Fig. 3D, E). In contrast, 5xFAD mice in the MST1 knockdown group showed a significant upward trend in both of the aforementioned indicators compared with the empty virus group. Track plots on the fifth day of training showed representative swimming paths for each group of mice (Fig. 3 F). Overall, these results suggest that the cognitive function of 6-month-old 5xFAD mice is significantly impaired, but that MST1 knockdown can notably improve their spatial learning and memory abilities. This indicates that MST1 knockdown alleviates cognitive deficits in 5xFAD mice.

### Knockdown of MST1 reduces Tau hyperphosphorylation and neurodegeneration in 5xFAD mice

**Fig. 4:**
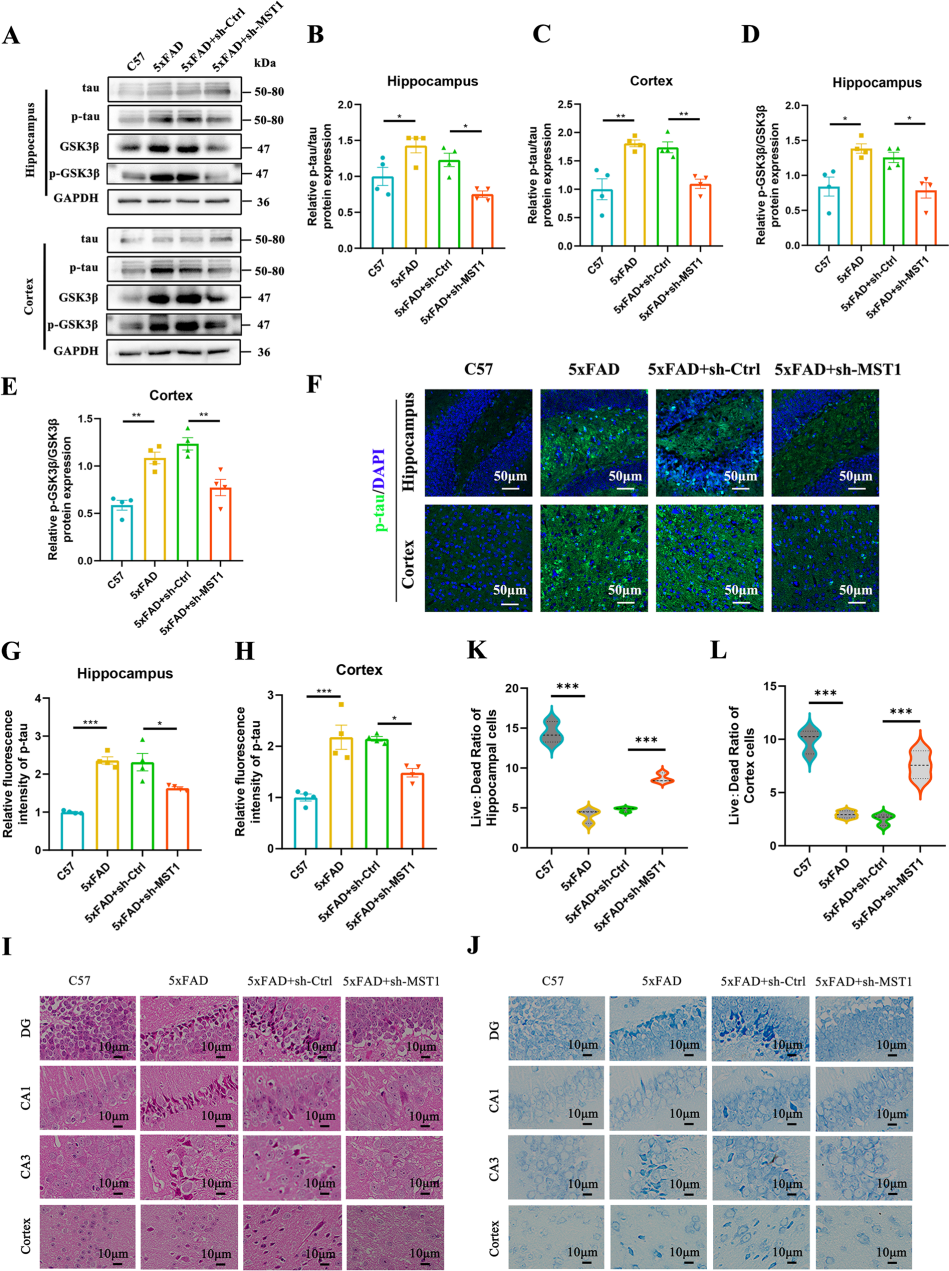
Knockdown of MST1 reduces tau hyperphosphorylation and neurodegeneration in 5xFAD mice. **A**–**E**. Representative Western blot images illustrating relative protein levels of total tau, p-tau (S396), total GSK3β, and p-GSK3β in hippocampal and cortical tissues (*n* = 4 mice per group). **F**–**H**. Representative immunofluorescence images and quantitative analysis of p-tau(S396) in hippocampal and cortical tissues (*n* = 4 mice per group; 2 sections/mouse; 3 fields/section; data averaged per mouse, original magnification: × 20, scale bar is 50 μm). **I**. Representative images of HE staining in the hippocampus (DG, CA1, and CA3 subregions) and cortical areas (*n* = 3 mice per group; 2 sections/mouse; local magnification: × 200, scale bar is 10 μm). **J**. Representative images of Nissl staining in the hippocampus (DG, CA1, and CA3 subregions) and cortical areas (*n* = 3 mice per group; local magnification: × 200, scale bar is 10 μm). K–L. The ratio of surviving to dead neurons in the hippocampus and cortex (*n* = 3 mice per group; 2 sections/mouse; 3 fields/section; data averaged per mouse). One-way ANOVA with Tukey’s multiple comparison test was employed. Data are expressed as mean ± SEM, *p < 0.05, **p < 0.01, ***p < 0.001

Hyperphosphorylation of Tau protein is one of the most prominent pathological features in the progression of AD, and the key role of GSK3β in AD progression lies in its involvement in the phosphorylation process of tau protein. To determine whether MST1 knockdown can alleviate tau protein pathological changes in 5xFAD mice, we performed Western blot and immunofluorescence experiments for verification. Western blot analysis showed that, compared with the control group, the p-tau/tau and p-GSK3β/GSK3β ratios were significantly increased in both the hippocampus (Fig. 4A, B, D and Fig S2A, B) and cortex (Fig. 4A, C, E) of 5xFAD mice. In contrast, compared with 5xFAD mice injected with empty vector virus, MST1 knockdown significantly reduced the p-tau/tau and p-GSK3β/GSK3β ratios in both the hippocampus (Fig. 4A, B, D) and cortex (Fig. 4A, C, E). Consistently, the analysis of immunofluorescence staining results showed that the level of p-tau in the hippocampus and cortex of 5xFAD mice with MST1 knockdown was significantly reduced (Fig. 4F, G, H). These results indicate that MST1 knockdown can effectively reduce the phosphorylation level of tau protein in 5xFAD mice.

HE staining (Fig. 4I and Fig. S2C) revealed normal morphology of hippocampal and cortical neurons in C57 mice, characterized by compact cell arrangement, abundant cytoplasm, uniform staining, and clear nuclear structure. In contrast, some neurons in the hippocampus and cortex of 5xFAD mice exhibited shrinkage, with sparse cell arrangement and deepened chromatin staining. Notably, after MST1 knockdown, the degree of neuronal damage in the hippocampus and cortex of 5xFAD mice was significantly reduced, and cell morphology improved partially. Similarly, Nissl staining (Fig. 4J and Fig. S2D) showed that neurons in C57 mice exhibited clear nuclei and a dense cellular arrangement. In contrast, neurons in the hippocampus and cortex of 5xFAD mice displayed pronounced morphological abnormalities, including sparse arrangement and cellular shrinkage, accompanied by a significant decrease in the live-to-dead neuron ratio (Fig. 4K, L) and a marked increase in the absolute number of dead neurons (Fig. S2E, F). Notably, compared with 5xFAD mice injected with empty vector virus, MST1 knockdown in 5xFAD mice markedly alleviated neuronal damage in both the hippocampus and cortex, as evidenced by a significant increase in the live-to-dead neuron ratio (Fig. 4K, L) and a substantial reduction in the absolute number of dead neurons (Fig. S2E, F). Overall, MST1 knockdown improves neuronal damage during disease progression in 5xFAD mice, thereby delaying neurodegeneration.

### MST1 knockdown alleviates glial cell activation and neuroinflammation in 5xFAD mice

**Fig. 5:**
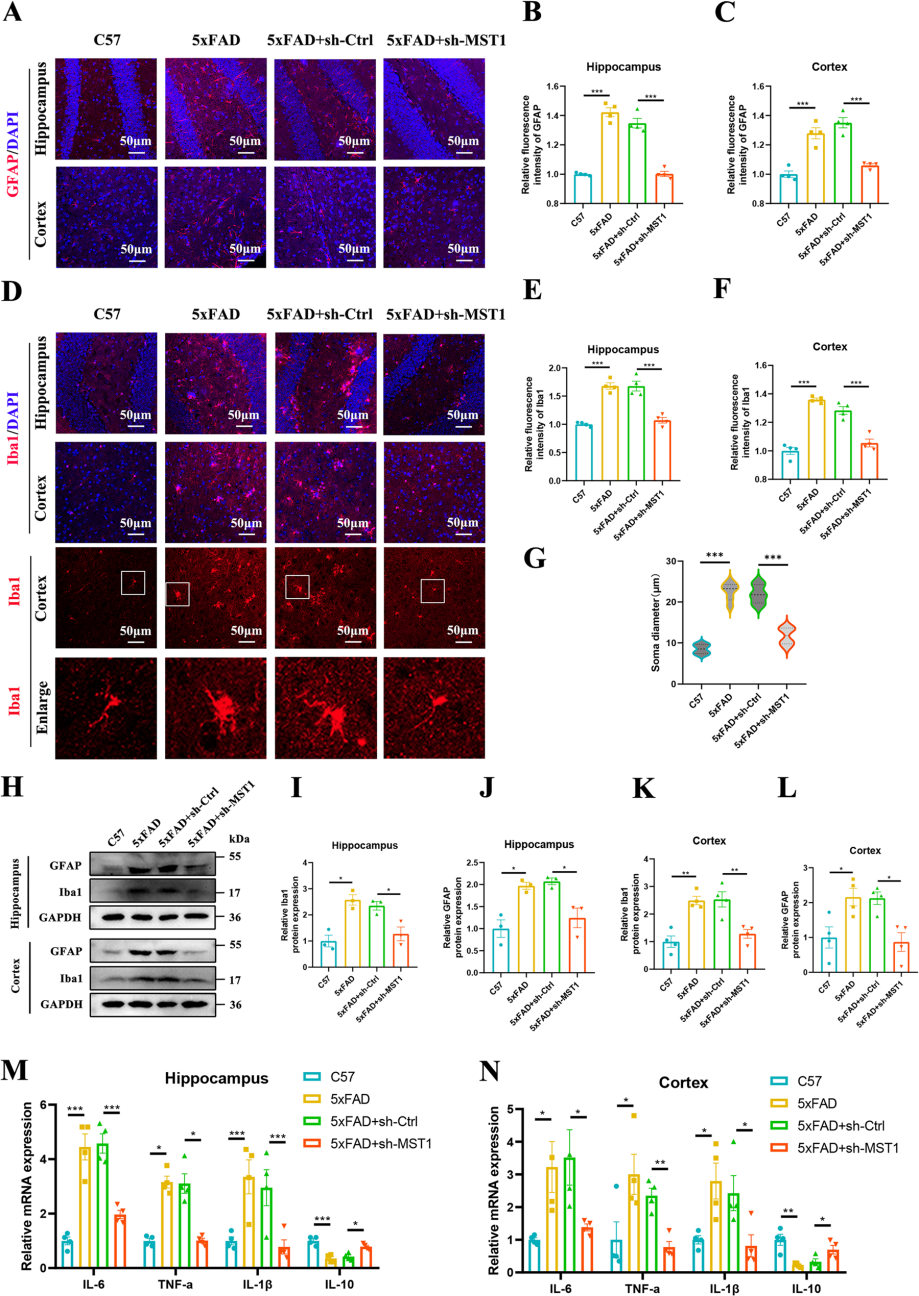
Knockdown of MST1 alleviates glial cell activation and neuroinflammation in 5xFAD mice. **A**–**C**. Representative immunofluorescence images and quantitative analysis of GFAP in hippocampal and cortical tissues. Mark astrocytes with the GFAP antibody (*n* = 4 mice; 2 sections/mouse; 3 fields/section; data averaged per mouse, original magnification: × 20, scale bar is 50 μm). **D**–**F**. Representative immunofluorescence images and quantitative analysis of Iba1 in hippocampal and cortical tissues. Mark microglia with the Iba1 antibody (*n* = 4 mice; 2 sections/mouse; 3 fields/section; data averaged per mouse, original magnification: × 20, scale bar is 50 μm). **G**. Morphological analysis of microglia reveals alterations in soma diameter (*n* = 6 mice per group). **H**–**L**. Representative Western blot images illustrating relative protein levels of GFAP (**H**, **J**, **L**) and Iba1 (**H**, **I**,** K**) in the hippocampus and cortex tissues (*n* = 3–4 mice per group). **M**, **N**. RT-qPCR revealed the expression levels of mRNA for inflammation-related factors (IL-6, TNF-α, IL-1β and IL-10) in hippocampal and cortical tissues (*n* = 4 mice per group). One-way ANOVA with Tukey’s multiple comparison test was employed. Data are expressed as mean ± SEM, *p < 0.05, **p < 0.01, ***p < 0.001

Glial cell activation is an important feature of AD progression. We used GFAP staining to observe astrocyte activation. Immunofluorescence results showed that, compared to C57 mice, GFAP expression was significantly increased in the hippocampus and cortex of 5xFAD mice; after MST1 knockdown, GFAP expression was significantly decreased in the hippocampus and cortex of 5xFAD mice (Fig. 5A, B, C). Iba1 is an F-actin-binding protein that is specifically expressed in microglia, and increased Iba1 expression can be used as a marker for microglial activation. Immunofluorescence staining of Iba1 showed that, compared to C57 mice, Iba1 expression was significantly increased in the hippocampus and cortex of 5xFAD mice, while in 5xFAD mice with MST1 knockdown, Iba1 expression was significantly decreased (Fig. 5D, E, F). Microglial morphology analysis revealed that the morphology of microglia in the brain tissue of 5xFAD mice changed from a ‘branched’ shape (resting state) to an ‘amoeboid’ shape (activated state), with enlarged cell bodies and increased cell body diameter (Fig. 5D, G). Western blot results showed that the expression trends of GFAP and Iba1 proteins in each group of mice were consistent with the immunofluorescence results (Fig. 5H, I, J, K, L). These results suggest that glial cells are activated in the brain tissue of 5xFAD mice and that MST1 knockdown alleviates this activation and stabilises the cells’ state.

Different states of neuroglial cells result in the production of various cytokines, including proinflammatory cytokines (IL-6, IL-1β and TNF-α) and anti-inflammatory cytokines (IL-10). To evaluate the effect of MST1 on cytokine levels in the brain tissue of 5xFAD mice, RT-qPCR analysis was performed. Compared with the C57 group, the results showed that the expression of proinflammatory cytokines (IL-6 mRNA, IL-1β mRNA and TNF-α mRNA) in the hippocampus and cortex of 5xFAD mice was significantly increased, while the expression level of the anti-inflammatory cytokine IL-10 mRNA was significantly decreased (Fig. 5M, N). Conversely, compared to 5xFAD mice injected with an empty virus, MST1 knockdown significantly reduced the expression of proinflammatory cytokines (IL-6 mRNA, IL-1β mRNA and TNF-α mRNA) in the hippocampus and cortex of 5xFAD mice, while significantly increasing the expression of the anti-inflammatory cytokine IL-10 mRNA (Fig. 5M, N). Based on these results, we conclude that MST1 knockdown reduces the expression of proinflammatory cytokines and increases the expression of anti-inflammatory cytokines, thereby alleviating AD related neuroinflammatory responses.

### Knockdown of MST1 promotes a shift in microglial activation from pro-inflammatory state toward an anti-inflammatory state

**Fig. 6:**
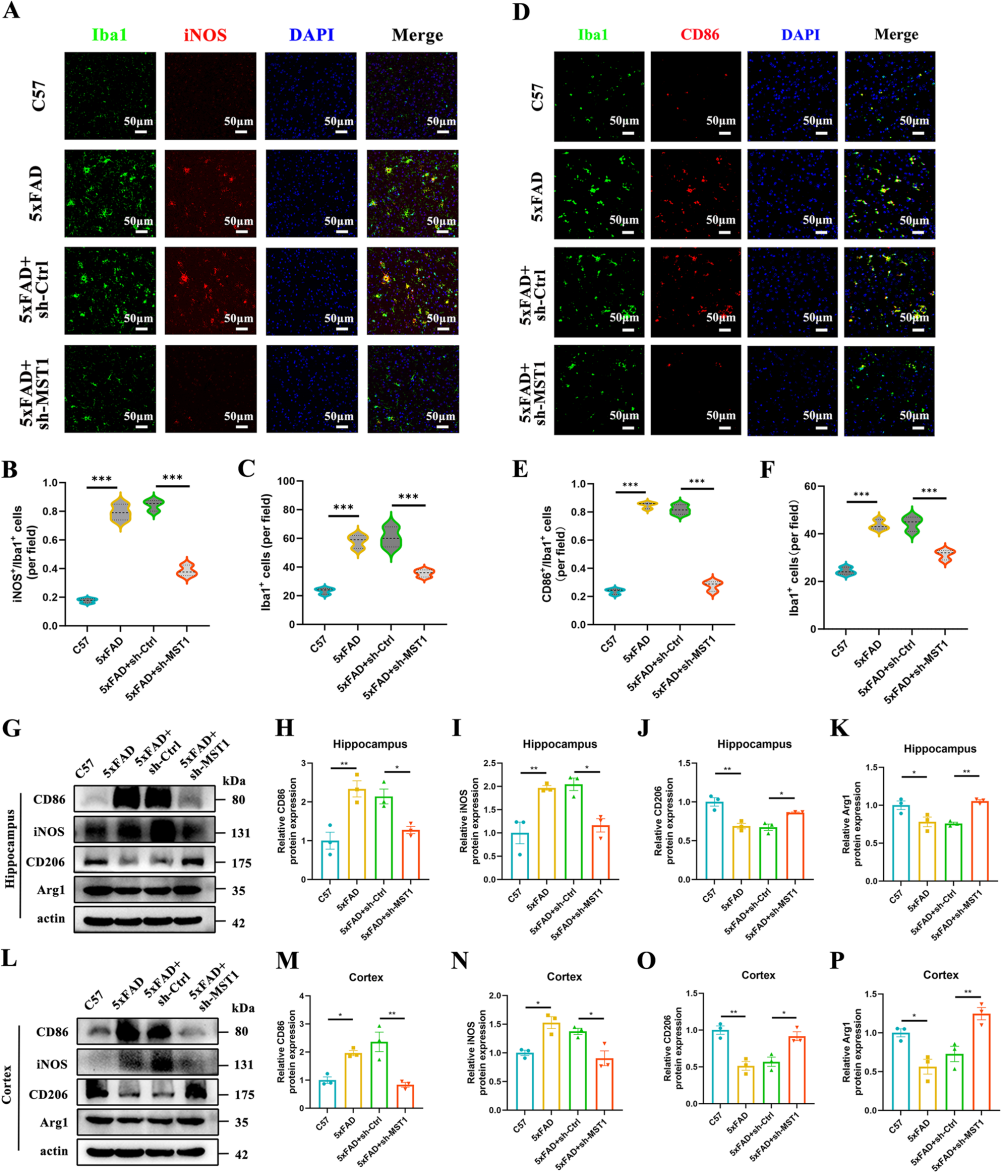
MST1 knockdown suppresses microglial activation. **A** Representative images of double immunofluorescence staining for Iba1 (green) and iNOS (red) in the cortex, with DAPI (blue) used for nuclear counterstaining (*n* = 3 mice; original magnification: × 40, scale bar is 50 μm). **B** The number of iNOS-positive cells/the number of Iba1-positive cells per field (*n* = 3 mice per group; 2 sections/mouse; 3 fields/section; data averaged per mouse). **C** The number of Iba1-positive cells per field (*n* = 3 mice per group; 2 sections/mouse; 3 fields/section; data averaged per mouse). **D** Representative images of double immunofluorescence staining for Iba1 (green) and CD86 (red) in the cortex, with DAPI (blue) used for nuclear counterstaining (*n* = 3 mice; original magnification: × 40, scale bar is 50 μm). **E** The number of CD86-positive cells/the number of Iba1-positive cells per field (*n* = 3 mice per group; 2 sections/mouse; 3 fields/section; data averaged per mouse). **F** The number of Iba1-positive cells per field (*n* = 3 mice per group; 2 sections/mouse; 3 fields/section; data averaged per mouse). **G**–**K**. Representative Western blot images illustrating relative protein levels of CD86, iNOS, CD206 and Arg1 in the hippocampus tissues (*n* = 3 mice per group). **L**–**P**. Representative Western blot images illustrating relative protein levels of CD86, iNOS, CD206, and Arg1 in the cortex tissues (*n* = 3 mice per group). One-way ANOVA with Tukey’s multiple comparison test was employed. Data are expressed as mean ± SEM, *p < 0.05, **p < 0.01, ***p < 0.001

In recent years, accumulating evidence has suggested that microglial activation is not a simple binary “polarization” but rather represents a continuum of functional states driven by microenvironmental cues and dynamically shaped across spatial, temporal, and pathological contexts. Within this spectrum, microglia can exhibit varying degrees of inflammatory and reparative/homeostatic features, which are often operationally described as pro-inflammatory microglia or anti-inflammatory microglia to indicate their predominant functional bias [40]. Inducible nitric oxide synthase (iNOS) is a well-recognized marker of microglial activation. Previous studies have demonstrated that CD86 and iNOS are predominantly expressed in pro-inflammatory microglia, whereas CD206 and Arg1 are mainly expressed in anti-inflammatory microglia in brain tissue. In this study, double immunofluorescence staining for Iba1 in combination with iNOS, CD86, Arg1, or CD206 was performed to evaluate the effects of MST1 deletion on microglial activation phenotypes. The results showed that, compared with control mice, the numbers of iNOS⁺/Iba1⁺ and CD86⁺/Iba1⁺microglia were significantly increased in the cortex of 5xFAD mice, whereas this increase was markedly attenuated following MST1 deletion (Fig. 6A, B, C, D, E and F). In contrast, compared with the 5xFAD group, MST1 deletion significantly increased the numbers of Arg1⁺/Iba1⁺and CD206⁺/ Iba1⁺ microglia in the cortex of 5xFAD mice. Together, these results indicate that MST1 knockdown suppresses pro-inflammatory microglial activation and promotes a shift toward an anti-inflammatory phenotype (Fig. S3A, B, C and Fig. S3D, E, F). Accordingly, we further performed Western blot analysis to examine the protein expression levels of pro-inflammatory microglial markers (CD86 and iNOS) and anti-inflammatory microglial markers (CD206 and Arg1), thereby indirectly reflecting the activation status and phenotypic profile of microglia in the brain. The results of the Western blot analysis showed that, compared with the control group, the protein expression of the pro-inflammatory microglia markers (CD86 and iNOS) was significantly increased in the hippocampus and cortex of 5xFAD mice, while the protein expression of the anti-inflammatory microglia markers (CD206 and Arg1) was significantly decreased. Further analysis revealed that, compared to 5xFAD mice injected with an empty virus, MST1 knockdown significantly reduced the protein expression of CD86 and iNOS in the hippocampus (Fig. 6G, H, I, J, K) and cortex (Fig. 6L, M, N, O, P), while significantly increasing the protein expression of CD206 and Arg1. In total, the results of double immunofluorescence staining and Western blot analyses indicated that MST1 deficiency suppresses the polarization of microglia toward a pro-inflammatory phenotype in the brains of 5xFAD mice.

### MST1 knockdown regulates the NLRP1/Caspase-1/GSDMD-N axis to alleviate microglial pyroptosis in AD

**Fig. 7:**
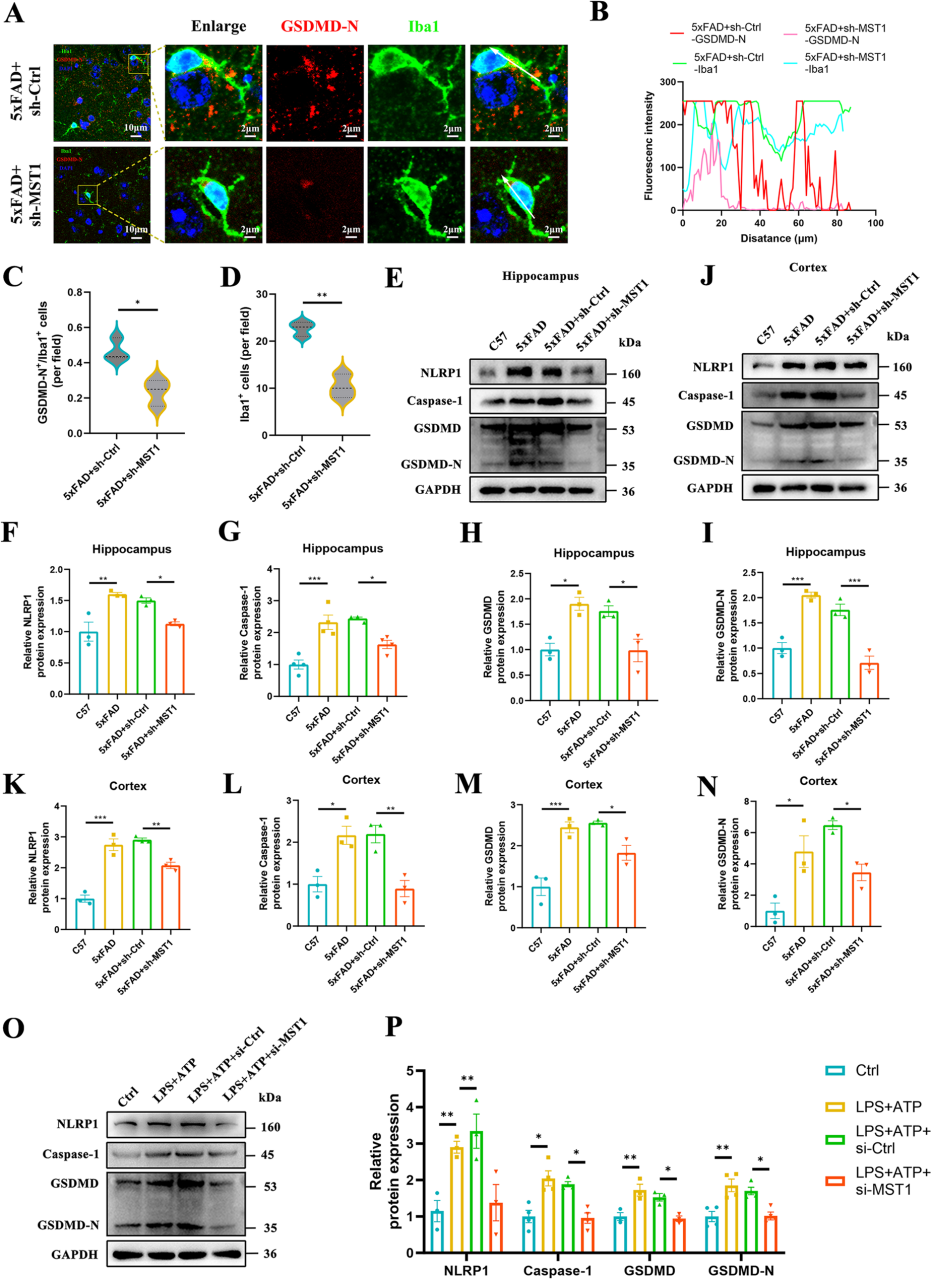
MST1 knockdown regulates the NLRP1/Caspase-1/GSDMD-N axis to alleviate microglial pyroptosis in AD. **A** Representative images of double immunofluorescence staining of Iba1 (green) and GSDMD-N (red) in the cortex. DAPI (blue) was used for nuclear counterstaining (*n* = 3 mice; Images were acquired using a confocal laser scanning microscope equipped with a 100× oil-immersion objective; scale bar is 10 μm; scale bar for enlarged insets is 2 μm). **B** Fluorescence intensity of Iba1 and GSDMD-N in cells indicated by the white arrows. **C** The number of GSDMD-N-positive cells/the number of iba1-positive cells per field (*n* = 3 mice per group; 1 sections/mouse; 3 fields/section; data averaged per mouse). **D** The number of iba1-positive cells per field (*n* = 3 mice per group; 1 sections/mouse; 3 fields/section; data averaged per mouse). **E**–**I**. Representative Western blot images illustrating relative protein levels of NLRP1, Caspase-1, GSDMD, and GSDMD-N in the hippocampus tissues (*n* = 3 mice per group). **J**–**N**. Representative Western blot images illustrating relative protein levels of NLRP1, Caspase-1, GSDMD, and GSDMD-N in the cortex tissues (*n* = 3 mice per group). **O**–**P**. Representative Western blot images illustrating relative protein levels of NLRP1, Caspase-1, GSDMD, and GSDMD-N in the BV2 cells induced by LPS and ATP. Two-tailed unpaired Student’s t-tests and One-way ANOVA with Tukey’s multiple comparison test were employed. Data are expressed as mean ± SEM, *p < 0.05, **p < 0.01, ***p < 0.001.

As is well-known, pyroptosis plays an important role in the progression of AD — microglial pyroptosis further exacerbates neuroinflammation; conversely, inhibiting pyroptosis-related signalling pathways may serve as an effective strategy for treating AD [41, 42]. In order to investigate the impact of MST1 on microglial pyroptosis, we examined and confirmed the alterations in the expression levels of pivotal pyroptosis-associated proteins (NLRP1, Caspase-1, GSDMD and GSDMD-N). Immunofluorescence co-staining revealed that GSDMD-N expression was significantly higher in the brains of 5xFAD mice than in the control group, and that this protein co-localised with Iba1 (a microglial marker). This indicates that microglial pyroptosis occurs in the brains of 5xFAD mice (Fig. 7A, B, C, D). Additionally, Western blot analysis revealed that the expression levels of pyroptosis-related proteins (NLRP1, Caspase-1, GSDMD and GSDMD-N) were significantly higher in the hippocampal (Fig. 7E, F, G, H, I) and cortical (Fig. 7J, K, L, M, N) tissues of 5xFAD mice than in C57 wild-type mice. Notably, the protein levels of NLRP1, Caspase-1, GSDMD and GSDMD-N in the hippocampal (Fig. 7E, F, G, H, I) and cortical (Fig. 7J, K, L, M, N) tissues of 5xFAD + sh-MST1 mice were all decreased compared with 5xFAD + sh-Ctrl mice.

Meanwhile, we treated BV2 microglia with LPS + ATP to induce pyroptosis and establish an in vitro model of microglial pyroptosis for AD (Fig. S4A). Subsequently, to further investigate the effect of this manipulation on pyroptosis in BV2 cells, we knocked down MST1 expression by transfecting siMST1 (Fig. S5A, B, C). Consistent with the in vivo experimental results, MST1 knockdown reversed the high expression of NLRP1, Caspase-1, GSDMD and GSDMD-N proteins induced by LPS + ATP in the BV2 cell model (Fig. 7O, P). Based on these results, we can conclude that AD progression is closely associated with pyroptosis. Furthermore, MST1 knockdown inhibits pyroptosis, which is expected to alleviate neuroinflammation and exert a neuroprotective effect.

### Proteomic analysis of hippocampal tissue in 5xFAD mice following MST1 knockdown

**Fig. 8:**
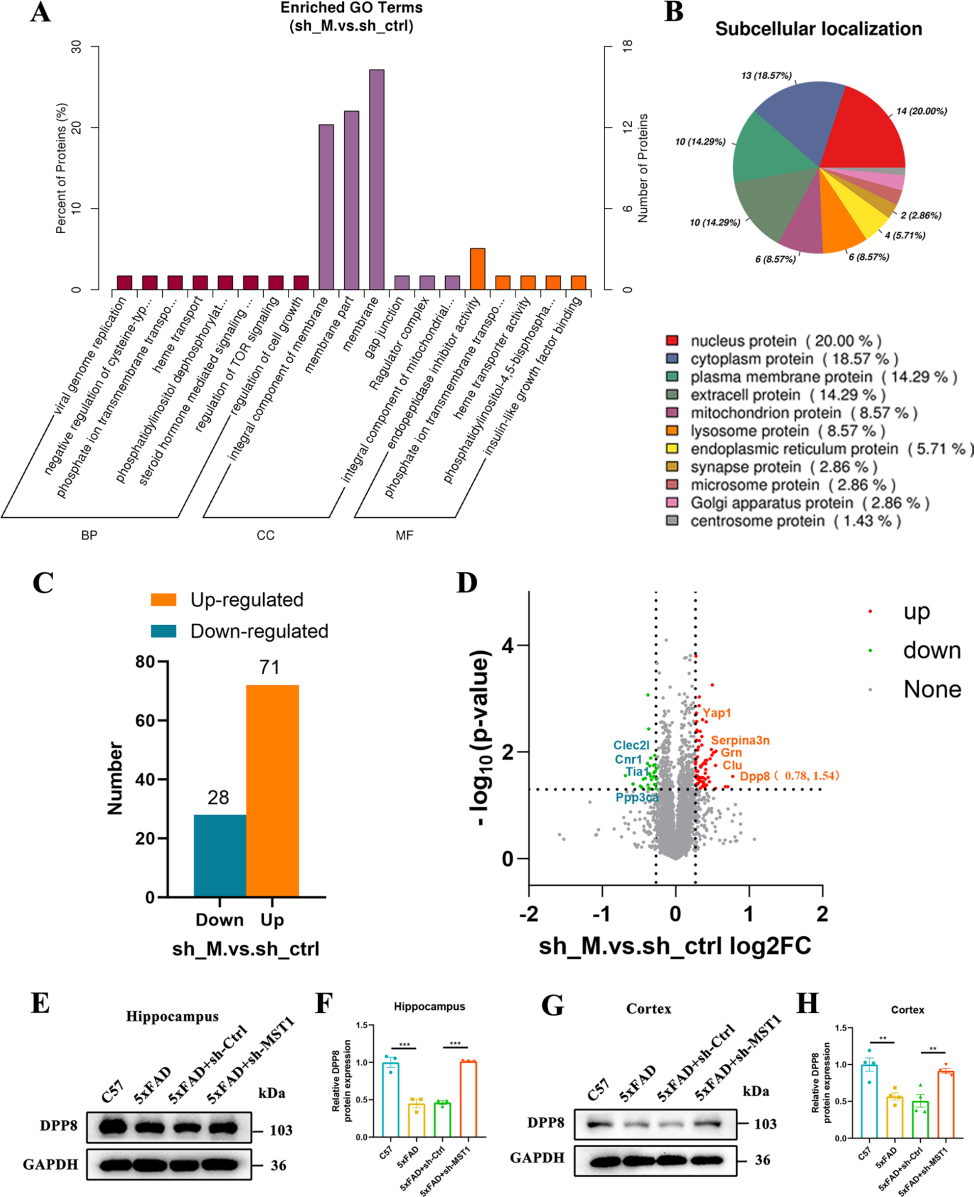
Proteomic analysis of differentially expressed proteins in the hippocampal tissue of 5xFAD mice with MST1 knockdown. We performed proteomic profiling and analysis of hippocampal tissues from the 5xFAD + sh-Ctrl and 5xFAD + sh-MST1 mouse groups, with three biological replicates per group. **A** Gene Ontology (GO) enrichment analysis of differentially expressed proteins. **B** Subcellular localization of differentially expressed proteins. **C** The number of differentially expressed proteins. **D** The abscissa represents the fold change of differentially expressed proteins (expressed as log_2_ value), and the ordinate represents the *p*-value (expressed as -log_10_ value). Proteins with no significant difference are shown in gray, upregulated proteins in red, and downregulated proteins in green. DPP8 (log_2_ FC: 0.78, –log_10_ *p*-value: 1.54). **E–F**. Representative Western blot images showing relative protein levels of DPP8 in the hippocampus tissues (*n* = 3 mice per group). **G–H**. Representative Western blot images showing relative protein levels of DPP8 in the cortex tissues (*n* = 3 mice per group). One-way ANOVA with Tukey’s multiple comparison test was employed. Data are expressed as mean ± SEM, **p < 0.01, ***p < 0.001

To further investigate the potential molecular mechanisms of MST1 in AD progression, TMT quantitative proteomics was used to detect protein expression in hippocampal tissue from 5xFAD + sh-Ctrl and 5xFAD + sh-MST1 mice. A total of 5,830 proteins were identified from six samples. Principal component analysis (PCA) showed that samples from each group exhibited good biological reproducibility, confirming the consistency between the biological replicates of the two groups (Fig. S6A). In addition, the heatmap combined with clustering analysis illustrated the relative abundance distribution of differentially expressed proteins (DEPs) across the samples (Fig. S6B). According to the criteria of *p* < 0.05 and |log_2_ (fold change) | ≥1.2 or ≤ 0.83, significantly DEPs were screened, and a total of 99 proteins with significant changes were finally selected. Gene Ontology (GO) enrichment analysis showed that these DEPs were primarily associated with processes such as cell growth regulation and immune-inflammatory regulation (e.g. TOR signalling and steroid hormone-mediated signalling) (Fig. 8A). Subcellular localization analysis indicated that the DEPs were mainly distributed in the nucleus, followed by the cytoplasm (Fig. 8B). Among the 99 DEPs, 71 proteins were up-regulated and 28 proteins were down-regulated (Fig. 8C). We then conducted a Kyoto Encyclopedia of Genes and Genomes (KEGG) database analysis, finding that the functions of these DEPs were involved in biological processes such as immune inflammation, cell communication and metabolism (Fig. S6C). Among DEPs, DPP8 was one of the proteins with relatively significant up-regulation (Fig. 8D). Finally, immunofluorescence analysis showed that MST1 knockdown significantly upregulated DPP8 expression in microglia in the brains of 5xFAD mice (Fig. S7A, B, C, D), further supporting the above findings. Additionally, Western blot results verified that the expression level of DPP8 in the hippocampus (Fig. 8E, F) and cortex (Fig. 8G, H) of 5xFAD mice was regulated by MST1.

### MST1 regulates LPS-induced inflammation and pyroptosis in BV2 cells by acting on the NLRP1/Caspase-1/GSDMD-N axis via DPP8

**Fig. 9:**
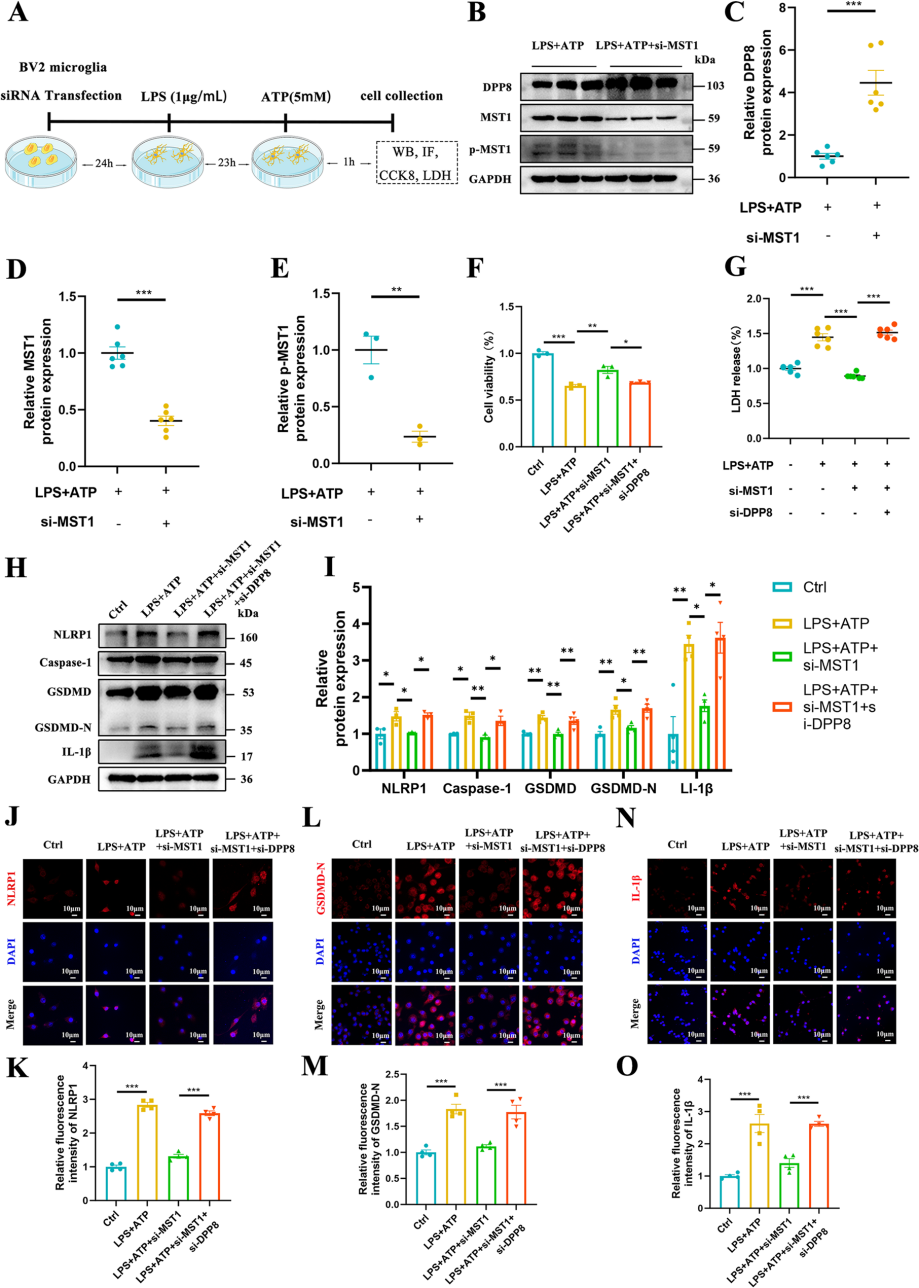
MST1 regulates LPS-induced inflammation and pyroptosis in BV2 cells by acting on the NLRP1/Caspase-1/GSDMD-N axis via Dpp8. **A**. Schematic diagram of cell experiment arrangement. **B**–**E**. Representative Western blot images illustrating relative protein levels of DPP8, MST1 and p-MST1(Thr183) (*n* = 3–6 biological replicates per group). **F**. CCK8 assay was used to detect the cell viability of BV2 cells after different interventions (*n *= 3 biological replicates per group). **G**. LDH assay kit was used to detect LDH release in BV2 cells after different interventions (*n* = 6 biological replicates per group). **H**-**I**. Representative Western blot images illustrating relative protein levels of NLRP1, Caspase-1, GSDMD, GSDMD-N, and IL-1β (*n* = 3–4 biological replicates per group). **J**–**O**. Representative immunofluorescence images and quantitative analysis of NLRP1 (J, K), GSDMD-N (L, M), IL-1β (**N**, **O**) in BV2 cells after different interventions (*n* = 3 biological replicates per group, original magnification: × 40, scale bar is 10 μm). Two-tailed unpaired Student’s t-tests and One-way ANOVA with Tukey’s multiple comparison test were employed. Data are expressed as mean ± SEM, *p < 0.05, **p < 0.01, ***p < 0.001

In the present study, proteomic analyses identified that MST1 regulates DPP8 expression in AD. Previous studies have demonstrated that inhibiting DPP8 can activate NLRP1 to induce pyroptosis and trigger macrophage pyroptosis via a caspase-1-dependent pathway [32, 33]. To investigate whether MST1 alleviates microglial pyroptosis in AD by influencing DPP8 to regulate the NLRP1/Caspase-1/GSDMD-N axis, we established a microglial pyroptosis model using LPS and ATP induction. We then conducted studies based on this model (Fig. 9A shows the experimental workflow). First, we revalidated the effect of MST1 on DPP8 in the LPS + ATP-induced BV2 cell model. Western blot results showed that the expression levels of MST1 and p-MST1 in BV2 cells were significantly decreased following transfection with siMST1 compared with the control group (Fig. 9B, D, E), while DPP8 protein expression was significantly increased (Fig. 9B, C). These results indicate that MST1 can also regulate DPP8 expression in an in vitro cell model.

Subsequently, we used siDPP8 RNA to reduce DPP8 expression (Fig. S8A, B, C), and investigated whether this could reverse the effect of MST1 downregulation on microglial pyroptosis. For this experiment, the cells were divided into four groups and subjected to different interventions: the control group (Ctrl), the ATP + LPS group, the ATP + LPS+siMST1 group and the ATP + LPS+siMST1 + siDPP8 group. Cell viability was measured in each group and the results of the CCK-8 assay showed that, compared with the control group, cell viability was significantly reduced after treatment with ATP + LPS; conversely, knockdown of MST1 improved cell viability, whereas further knockdown of DPP8 led to a significant decrease in cell viability. These results indicate that the protective effect of MST1 on cells can be reversed by DPP8 downregulation (Fig. 9F). Subsequently, we evaluated the degree of pyroptosis by detecting the level of LDH released. Compared with the control group, LPS + ATP treatment significantly increased LDH release in the culture medium. In contrast, LDH release decreased markedly after MST1 downregulation. Further inhibition of the DPP8 protein led to re-elevation of LDH release (Fig. 9G). These results suggest that MST1 downregulation reduces BV2 cell cytotoxicity by decreasing LDH release, whereas DPP8 downregulation reinduces the release of cytotoxic substances.

Additionally, we determined the expression levels of apoptosis-related proteins via Western blot analysis. The results showed that, following LPS + ATP treatment, the expression levels of pyroptosis-related proteins (NLRP1, Caspase-1, GSDMD, GSDMD-N and IL-1β) increased significantly. In contrast, MST1 knockdown reduced the expression levels of these proteins and further DPP8 knockdown reversed the decrease in expression caused by MST1 knockdown (Fig. 9H, I). The results of immunofluorescence staining for NLRP1(Fig. 9J, K), GSDMD-N (Fig. 9L, M), and IL-1β (Fig. 9N, O) were consistent with the Western blot analysis trend. Together, these findings suggest that MST1 can regulate pyroptosis in LPS + ATP-stimulated BV2 cells via the DPP8/NLRP1/Caspase-1/GSDMD-N axis. They also illustrate the critical role of DPP8 in the pyroptotic process of BV2 cells.

### Knockdown of MST1 inhibits the neurotoxicity of HT22 cells induced by activated BV2 cells, and this effect can be reversed by DPP8

**Fig. 10:**
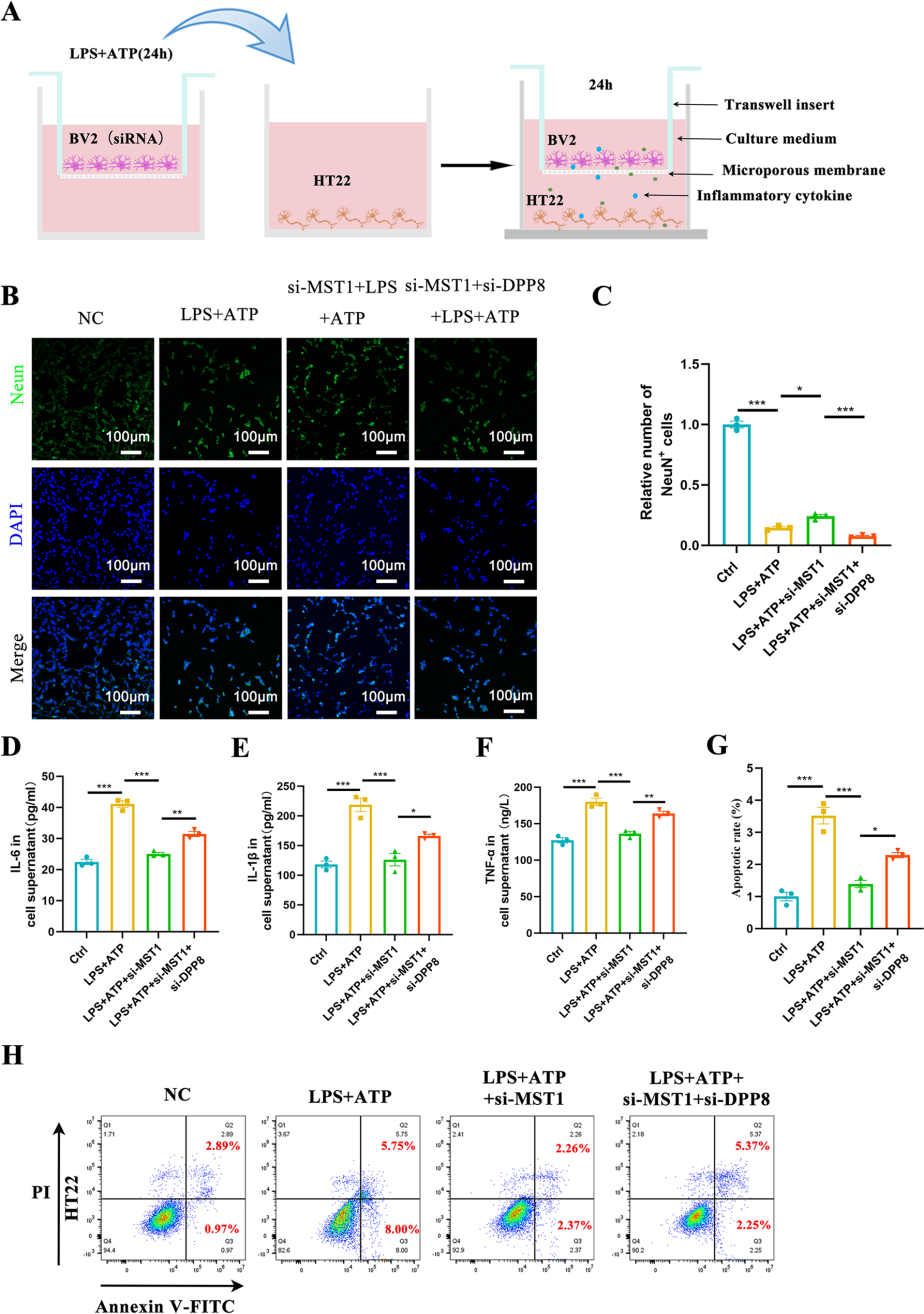
Knockdown of MST1 inhibits the neurotoxicity of HT22 cells induced by activated BV2 cells, and this effect can be reversed by DPP8. **A**. Schematic diagram of the BV2-HT22 cell interaction model. **B**-**C**. Representative immunofluorescence images and quantitative analysis of Neun (a neuronal marker) illustrating the state and number of HT22 cells after 24 h of co-cultivation (*n* = 3 biological replicates per group, original magnification: × 10, scale bar is 10 μm). **D**–**F**. Levels of proinflammatory cytokines (IL-6, IL-1β, and TNF-α) in the supernatant of HT22 cells after 24 h of co-cultivation (*n* = 3 biological replicates per group). **G**–**H**. Flow cytometry analysis cells apoptosis of HT22 cells after 24 h of co-cultivation in different groups (*n* = 3 biological replicates per group). One-way ANOVA with Tukey’s multiple comparison test was employed. Data are expressed as mean ± SEM, *p < 0.05, **p < 0.01, ***p < 0.001

The functional status of microglia (such as activation, pyroptosis, and phenotypic transformation) exerts an impact on the survival and function of neurons [27]. To simulate the in vivo interaction between microglia and neurons, this study conducted a co-culture experiment using BV2 and HT22 cells. The aim was to further evaluate the effect of altered MST1 and DPP8 expression on HT22 cell function by regulating pyroptosis in BV2 cells. The experimental procedure involved seeding normal BV2 cells, MST1-knockdown BV2 cells and BV2 cells with dual knockdown of MST1 and DPP8 separately in the upper chamber of the co-culture system. These cells were then stimulated with LPS + ATP for 24 h and subsequently cultured with HT22 cells in the lower chamber for a further 24 h (Fig. 10A shows the detailed co-culture workflow).

Immunofluorescence results showed that, compared with the control group, the number of HT22 cells in the LPS + ATP stimulation group was significantly reduced. In contrast, after MST1 knockdown, both the state and number of HT22 cells improved. However, in the group with dual MST1 and DPP8 knockdown, the number of HT22 cells decreased again (Fig. 10B, C). An ELISA assay was then performed to detect the levels of inflammatory cytokines in the co-culture supernatant. Compared with the control group, the levels of inflammatory cytokines (IL-6, IL-1β, and TNF-α) in the supernatant increased significantly after LPS + ATP stimulation. MST1 knockdown reduced the expression of these pro-inflammatory cytokines; however, in the co-culture supernatant of cells with dual knockdown of MST1 and DPP8, the release of these pro-inflammatory cytokines increased again (Fig. 10D, E, F). Finally, flow cytometry results for detecting HT22 cell apoptosis indicated that, compared with the control group, HT22 cell apoptosis in the LPS + ATP group was significantly increased. After MST1 knockdown, HT22 cell apoptosis was significantly alleviated, while after dual knockdown of MST1 and DPP8, HT22 cell apoptosis increased further (Fig. 10G, H). These results confirm that MST1 affects the release of inflammatory cytokines and the apoptosis of neuronal cells (HT22 cells) by regulating microglia, and that this regulatory effect can be reversed by downregulating DPP8.

## Discussion

Recent studies have indicated that microglia-mediated neuroinflammation is not only a key participant in the pathological progression of AD but also more likely a core pathogenic mechanism driving the onset and development of the disease [43]. Microglial pyroptosis is triggered by chronic inflammatory stimulation, and excessive pyroptosis further exacerbates inflammatory responses, forming a vicious cycle that ultimately leads to neuronal damage and accelerates the progression of AD [44]. In this study, we first confirmed that the activation of MST1 is closely associated with the progression of AD. More importantly, we discovered a novel mechanism by which MST1 can regulate microglial pyroptosis in AD for the first time, demonstrating its ability to inhibit pyroptosis via the DPP8/NLRP1/Caspase-1/GSDMD-N pathway. This mechanism breaks the vicious cycle of neuroinflammation in AD and alleviates neuronal damage. Therefore, targeting MST1 shows great promise for the development of AD treatments.

Alzheimer’s disease, a neurodegenerative disorder of the nervous system, has an incompletely elucidated pathogenesis to date. More problematically, the current diagnostic tools and therapeutic approaches for AD are relatively limited. Therefore, in-depth exploration of its pathogenesis and development of effective diagnostic and therapeutic strategies have become urgent medical challenges. Previous studies have shown that the total MST1 expression in the peripheral blood of AD patients is decreased and negatively correlated with patient age, as well as being positively correlated with MMSE score [45]. Other studies have confirmed that MST1 can be activated by various stress signals during disease progression and that this activation process is accompanied by autophosphorylation at multiple sites. Among these, phosphorylation at the Thr183 site is crucial for the functional activation of MST1 [46]. In this study, we found that the level of p-MST1 (Thr183) in the peripheral blood of AD patients was significantly higher than that in healthy controls. Further analysis revealed that this indicator was positively correlated with patient age and peripheral blood p-tau181 levels, and negatively correlated with MMSE and MoCA scores. ROC curve analysis showed that p-MST1 displays good sensitivity and specificity, indicating that it may serve as a candidate biomarker for AD diagnosis following further validation. Taken together with previous studies on the total expression and activation mechanism of MST1, these results suggest that MST1 may be activated into p-MST1 through phosphorylation and thus participate in the progression of AD. To verify the expression and activation status of MST1 multidimensionally, we systematically detected changes in the expression of MST1 and p-MST1 in 6-month-old 5xFAD mice and in BV2 cell models induced by Aβ_1−42_. In animal experiments, there was no significant change in total MST1 expression in 5xFAD mice compared with control mice. However, the expression level of p-MST1 was significantly increased in both hippocampal and cortical tissues, which is consistent with previous research reports [21, 47]. Notably, previous studies have not clearly clarified the expression of p-MST1 in the microglia of AD models. Our study confirmed that p-MST1 expression is increased in the microglia of AD models. Similarly, in the Aβ_1−42_-induced BV2 cell model, p-MST1 expression gradually increased with rising Aβ_1−42_ concentrations.

To investigate the potential role of MST1 in Alzheimer’s disease (AD), we employed intracerebroventricular injection to deliver AAV-shMST1 driven by a microglia-specific promoter, thereby achieving microglia-specific knockdown of MST1 in the hippocampal and cortical regions of mice. The Morris water maze test showed that MST1 knockdown significantly improved the spatial learning and memory abilities of 5xFAD mice. It is known that 5xFAD mice exhibit typical pathological features, including excessive production of Aβ42 and enhanced phosphorylation of tau at the Ser396 site [48]. However, previous studies have confirmed that MST1 has no significant effect on Aβ-related pathological processes [21, 47]. Consequently, we shifted the focus of our research to the regulatory role of MST1 in tau phosphorylation. Our results showed that, following MST1 knockdown, total tau protein expression remained largely unchanged, while the level of p-tau decreased significantly. 5xFAD is mainly an amyloid model and cannot fully simulate human tau pathology, so these findings are more likely to represent early changes in tau under amyloid background rather than complete tau pathology. Further analysis via HE staining and Nissl staining confirmed that MST1 knockdown could effectively rescue neuronal damage in the cerebral cortex and hippocampus, and promote the morphological reconstruction of neurons. This pathological improvement trend was consistent with the enhancement of cognitive function observed in the aforementioned behavioural experiments. These results confirm that MST1 knockdown alleviates neurodegeneration in 5xFAD mice, thereby improving their cognitive function. However, the present study did not systematically analyze the spatial distribution of AAV-infected microglia in relation to pathological regions, which represents a limitation of this work while also providing a valuable direction for future research.

The progression of AD is closely associated with excessive neuroinflammation. As the core immune effector cells in the central nervous system, astrocytes and microglia, upon activation, release a large amount of pro-inflammatory factors, thereby driving the progression of AD [49, 50]. Numerous studies have demonstrated that microglia play a key role in the progression of AD by regulating inflammation, modifying synapses, phagocytosing and undergoing phenotypic transformation [10, 51, 52]. Microglia-mediated neuroinflammation is well known to be a ‘double-edged sword’ in AD, being closely related to its two polarising phenotypes with diametrically opposed functions. M1 microglia exhibit a significantly enlarged and rounded cell body and release pro-inflammatory mediators such as IL-6, TNF-α and IL-1β, triggering neurotoxic damage. In contrast, M2 microglia secrete anti-inflammatory mediators such as IL-4 and IL-10 and initiate anti-inflammatory responses, thereby exerting neuroprotective effects [53–55]. Recent studies have emphasized that the traditional M1/M2 dichotomy is insufficient to capture the dynamic plasticity of microglia in vivo. Moreover, concerns regarding the excessive fragmentation of microglial subpopulations defined in single-cell RNA sequencing studies further suggest that microglial states may exist along a continuous spectrum [56]. In the present study, we focused primarily on the classical pro-inflammatory or anti-inflammatory states, which represents a limitation in fully characterizing the broader phenotypic landscape of microglia. In the current report, we first observed that astrocytes and microglia were significantly activated in the hippocampal and cortical tissues of 5xFAD mice. The mRNA expression levels of the pro-inflammatory cytokines IL-6, TNF-α and IL-1β were markedly increased, while the mRNA expression of the anti-inflammatory cytokine IL-10 was significantly decreased. This suggests a typical inflammatory imbalance in the brains of AD model mice. However, these pathological changes were significantly alleviated following MST1 knockdown. The activation of glial cells in the hippocampus and cortex of 5xFAD mice was effectively inhibited and the mRNA expression levels of pro-inflammatory cytokines (IL-6, TNF-α, and IL-1β) were notably reduced. Meanwhile, the mRNA expression of the anti-inflammatory cytokine IL-10 was significantly increased. Previous studies have reported that Hippo/MST1 signalling is involved in regulating microglial activation following acute cerebral ischaemia-reperfusion injury [26]. Further analysis of microglial phenotypes revealed that, in the brains of 5xFAD mice, microglia were predominantly polarised towards the pro-inflammatory M1 phenotype, characterised by the presence of CD86 and iNOS. In contrast, knockdown of MST1 was found to significantly promote the conversion of M1-polarised microglia to the anti-inflammatory and reparative M2 phenotype, characterised by the presence of CD206 and Arg1. Previous studies have indicated that MST1 can mediate IFN-γ-induced microglial apoptosis [57]. More recent studies have also found that overactivated microglia in AD undergo pyroptosis, which is a typical pro-inflammatory form of programmed cell death. Microglial pyroptosis and cell polarisation have a synergistic effect, amplifying the intracerebral inflammatory response and exacerbating neuronal damage [41]. Consistent with previous reports, we observed pyroptotic microglia in the hippocampus and cortex of 5xFAD mice, as evidenced by significantly increased expression of pyroptosis-related proteins (NLRP1, caspase-1, and GSDMD). Interestingly, MST1 knockout significantly down-regulated the expression of these pyroptosis-related proteins. This conclusion was also verified in cellular experiments: in the LPS + ATP-induced pyroptosis model of BV2 cells, MST1 knockdown similarly inhibited the expression of these proteins significantly. It should be noted that the LPS plus ATP stimulation paradigm is not sensor-specific, and therefore the potential contribution of the NLRP3 inflammasome cannot be excluded under these experimental conditions. Taken together, these results confirm that MST1 knockdown exerts neuroprotective effects through multiple pathways, including the inhibition of glial cell activation, the alleviation of neuroinflammation, the regulation of the polarization of microglia towards an anti-inflammatory phenotype and the reduction of microglial pyroptosis in 5xFAD mice.

To further explore the potential molecular mechanism of MST1 in the progression of AD, we employed TMT quantitative proteomics to analyse differences in protein expression in the hippocampal tissue of 5xFAD + sh-Ctrl and 5xFAD + sh-MST1 mice. Bioinformatics analysis revealed that reduced MST1 expression in the hippocampus of 5xFAD mice induced the expression of differential proteins, mainly enriched in processes related to cell growth, metabolism and immune inflammation. This finding is highly consistent with the results of our previous transcriptomic study [21]. Furthermore, previous studies have confirmed the important role of MST1/2 in inflammatory and immune processes [58, 59]. Additionally, other studies have reported that inhibiting MST1/2 can affect the repair process after myocardial infarction by regulating macrophage responses [60]. Among the differentially expressed proteins detected, we found that the DPP8 protein was significantly upregulated in the 5xFAD + sh-MST1 group. Previous studies have reported that DPP8 plays a key role in various diseases and can mediate various physiological and pathological processes, such as immune regulation, inflammatory response, and cancer cell biology [61–63]. Mechanistically, multiple recent studies have confirmed that, as a serine protease, DPP8 inhibitors can activate the inflammasome sensor protein Nlrp1b [29, 64], which in turn mediates pyroptosis by activating pro-caspase-1 [32, 33]. Furthermore, it has been suggested that DPP8/9 can act as an intracellular immune checkpoint, inhibiting Nlrp1b and the innate immune system [33]. Notably, a previous large-scale GWAS analysis of memory performance and cognitive decline in four aging cohorts identified the DPP8 gene on chromosome 15 as a novel gene associated with memory decline [65]. This finding suggests that DPP8 may play a role in the progression of AD. Validation of the proteomics results revealed that DPP8 expression decreased in the hippocampus and cortex of 5xFAD mice, whereas it increased significantly following MST1 knockdown. This result further supports the proteomics findings. Based on this evidence, we hypothesise that MST1 can regulate the NLRP1/Caspase-1/GSDMD-N axis by affecting DPP8 expression, thereby regulating AD-related microglial pyroptosis.

To verify the above hypothesis, we conducted experiments using using BV2 cells pyroptosis model induced by LPS + ATP. First, we confirmed that knocking down MST1 also promoted DPP8 expression in the cellular model, consistent with results from the 5xFAD mouse model. Subsequent experiments showed that LPS + ATP treatment and promoted LDH release. It also markedly increased the expression levels of proteins such as NLRP1, Caspase-1, GSDMD, GSDMD-N and IL-1β. These results confirmed that LPS + ATP successfully induced pyroptosis. Further studies revealed that, following MST1 knockdown, all the aforementioned pyroptosis-related indicators decreased significantly; however, when DPP8 was knocked down in addition to MST1 knockdown, these indicators increased again. These results suggest that DPP8 acts as a downstream protein of MST1 and that inhibition of DPP8 can reverse the protective effect of MST1 knockdown, thereby promoting pyroptosis in BV2 cells. Microglial pyroptosis is a type of pro-inflammatory programmed cell death whose core feature is the formation of pores in the cell membrane by the pyroptosis effector protein GSDMD-N. This leads to the ‘passive leakage’ of large quantities of proinflammatory cytokines, enzymes, and toxic molecules from the cell to the surrounding brain tissue microenvironment, damaging neurons in the process [15]. To simulate this pathological process in vivo, the cell co-culture experiment in this study found that, after co-culturing with BV2 cells in an LPS- and ATP-induced pyroptotic state, the status of HT22 neuronal cells deteriorated significantly. The level of proinflammatory cytokines in the culture medium increased markedly and the number of apoptotic cells rose significantly. Further intervention experiments revealed that MST1 knockdown reduced the release of inflammatory cytokines from BV2 cells, thereby protecting HT22 cells. However, this protective effect was reversed when DPP8 was knocked down in addition to MST1. These results confirm that inhibiting MST1 can increase DPP8 expression, thereby preventing the activation of the NLRP1/Caspase-1/GSDMD-N signalling axis and reducing the occurrence of microglial pyroptosis to achieve a neuroprotective effect.

This study has several limitations that should be acknowledged. First, the sample size of the clinical data is relatively small, which may increase susceptibility to false-positive findings; therefore, we plan to validate these results by collecting multicenter clinical data in future studies. Second, the interventional role of DPP8 in in vivo animal models, as well as the regulatory effects of complex inflammatory molecules involved in pyroptosis, require further investigation. In addition, given the increasing recognition of sex differences in microglial activation, hormone–immune interactions, and Alzheimer’s disease risk, the exclusive use of male mice in this study may limit the generalizability of our findings to the female population.

An important point to clarify is that the primary aim of this study was to elucidate the molecular mechanisms by which MST1 regulates microglial pyroptosis and its contribution to Alzheimer’s disease–related pathology, rather than to establish a model that fully recapitulates the entire course of human AD. The 5xFAD mouse model was selected because it reliably reproduces core AD pathological features, particularly amyloid-β deposition, and its early plaque formation facilitates the investigation of microglial dysfunction at the initial stages of pathology. The use of BV2 cells was based on their stable genetic background and high experimental reproducibility, which make them well suited for mechanistic studies involving molecular manipulations such as gene silencing or overexpression. Nevertheless, the rapid pathological progression observed in 5xFAD mice does not fully mirror the chronic development of human AD, and the immortalized nature of BV2 cells may lead to functional differences compared with primary microglia. Future studies will therefore incorporate primary human microglia (or iPSC-derived microglia) and disease models that more closely reflect the human AD trajectory, in order to further enhance the translational relevance of our findings.

It should be noted that the evidence for microglial pyroptosis in vivo in the present study was primarily based on immunofluorescence colocalization analysis, and the expression of related proteins was further evaluated at the whole-brain tissue level. Although these findings provide supportive evidence for the occurrence of microglial pyroptosis, future studies will benefit from combining microglial isolation and purification with quantitative assays such as Western blotting to further strengthen the cell-type specificity of our conclusions and to gain deeper insight into the underlying molecular mechanisms.

## Conclusions

This study demonstrated that, during the pathological progression of Alzheimer’s disease (AD), MST1 is activated into the p-MST1 form and participates in the progression of the disease. Preliminary analysis of clinical data suggests that p-MST1 could be a promising candidate biomarker that requires further validation for assisting in AD diagnosis. At a mechanistic level, the study confirmed that MST1 can regulate the inflammatory response, the polarization state and the pyroptosis process of microglia in AD models and identified DPP8 as an MST1 downstream effector protein. In summary, inhibiting MST1 can increase the expression of DPP8, which in turn inhibits the activation of the NLRP1/Caspase-1/GSDMD-N signalling axis. This reduces microglial pyroptosis and maintains homeostasis, ultimately exerting an AD neuroprotective effect.

## Supplementary Information

Supplementary Material 1.

Supplementary Material 2.

## Data Availability

Data presented in this study are available from the corresponding author upon reasonable request.

## Abbreviations

AD: Alzheimer’s disease
Aβ: β-amyloid
MST1: Mammalian ste20-like kinase 1
p-MST1: phosphorylated MST1
DPP8: Recombinant dipeptidyl peptidase 8
AAV: adeno-associated virus
MMSE: Mini-mental state examination
MoCA: Montreal cognitive assessment
siRNAs: Small interfering RNAs
ROC: Receiver operating characteristic curve
DEPs: differentially expressed proteins
