# Supplementary material for: MST1 promotes microglial pyroptosis and neuroinflammation in alzheimer’s disease by regulating the novel DPP8/NLRP1/Caspase-1/GSDMD-N axis
Source: J Neuroinflammation. 2026 Feb 13;23:95. doi: 10.1186/s12974-026-03732-3 (PMC13005356; doi:10.1186/s12974-026-03732-3)
Supplement: Supplementary file 1 — Supplementary Material 1. [file 12974_2026_3732_MOESM1_ESM.pdf]

Figure. 2A

Hippocampus

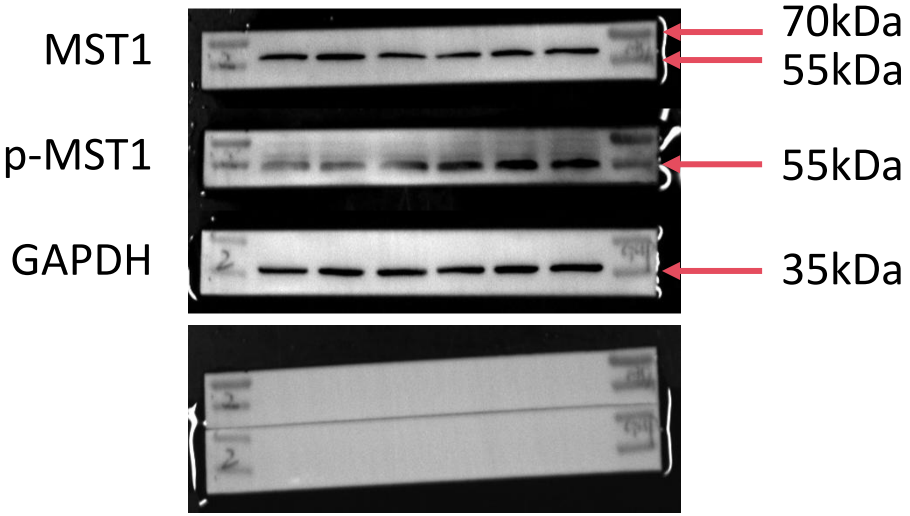

Figure. 2C

Cortex

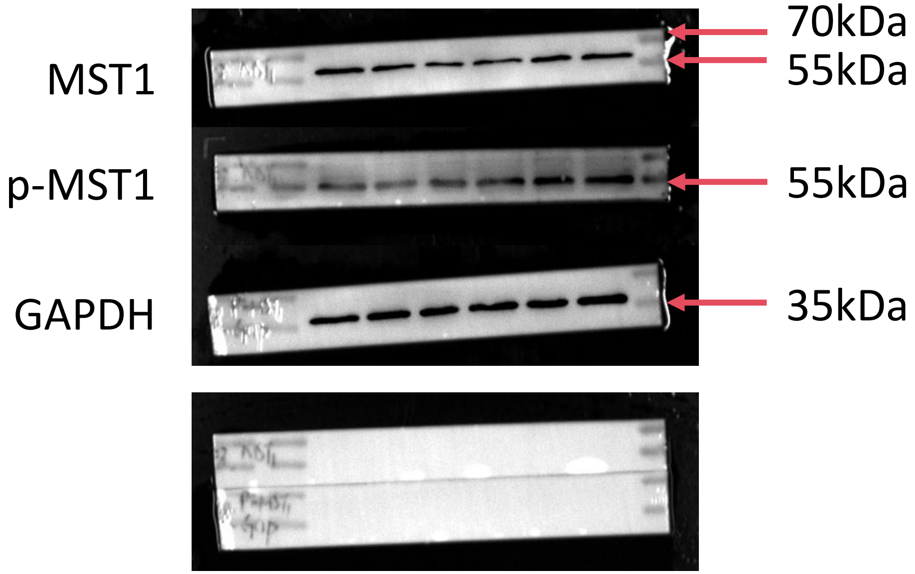

Figure. 2M

Bv2

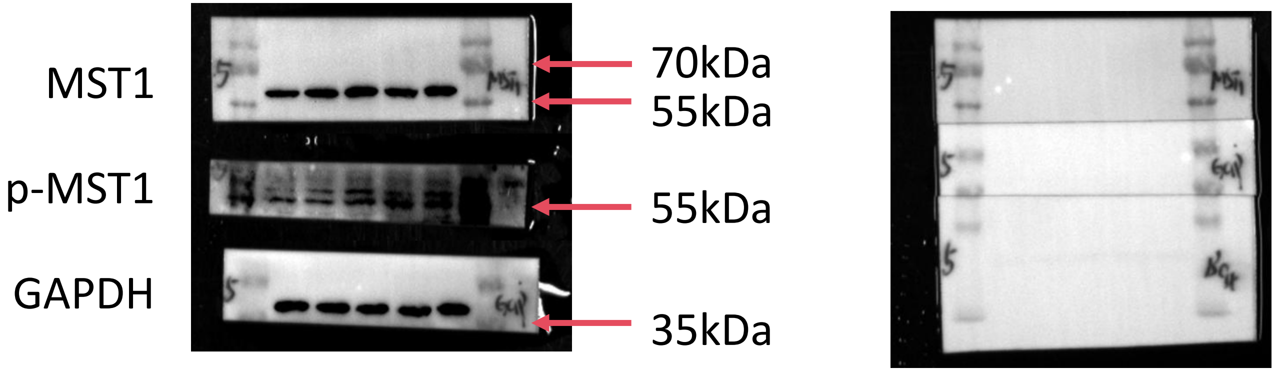

Figure. S1B

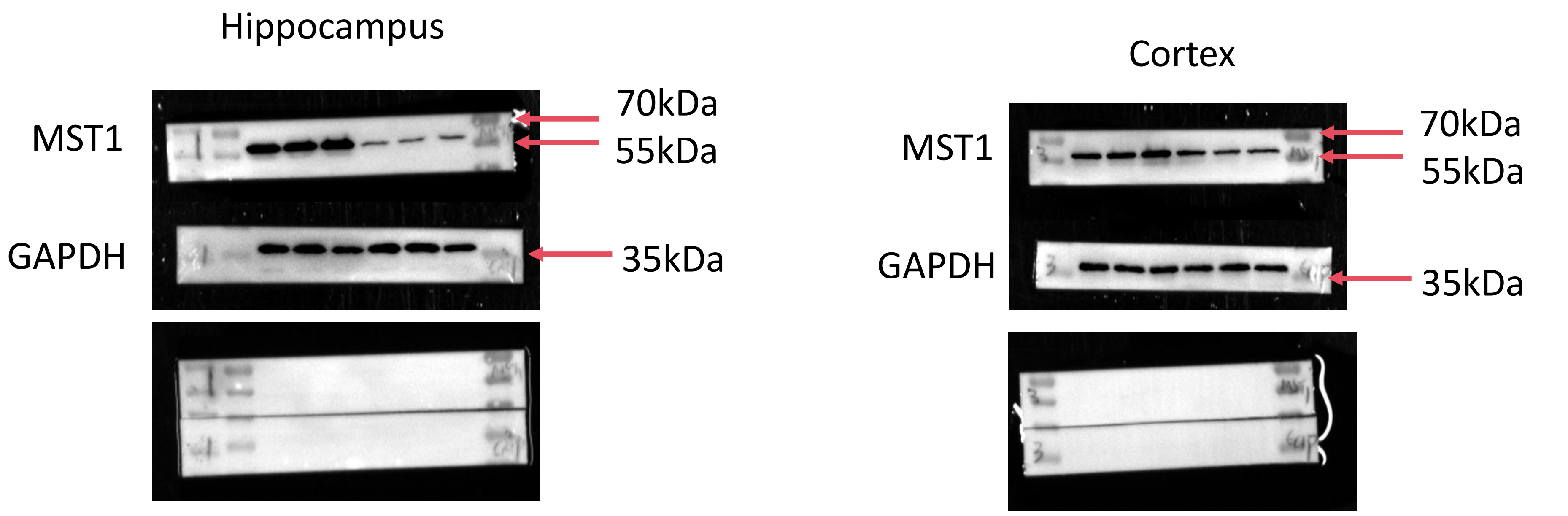
Hippocampus
MST1
GAPDH
70kDa
55kDa
35kDa
Cortex
MST1
GAPDH
70kDa
55kDa
35kDa

Figure. 4A (Hippocampus)

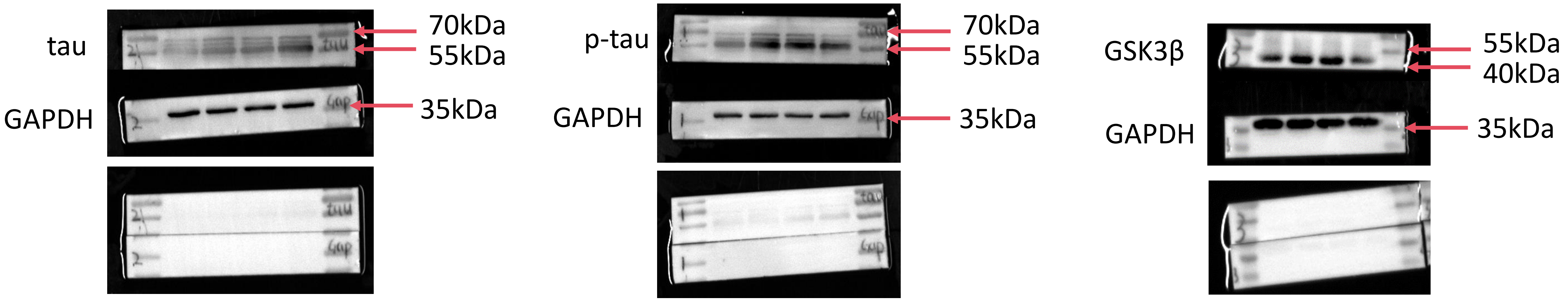

Figure. 4A (Cortex)

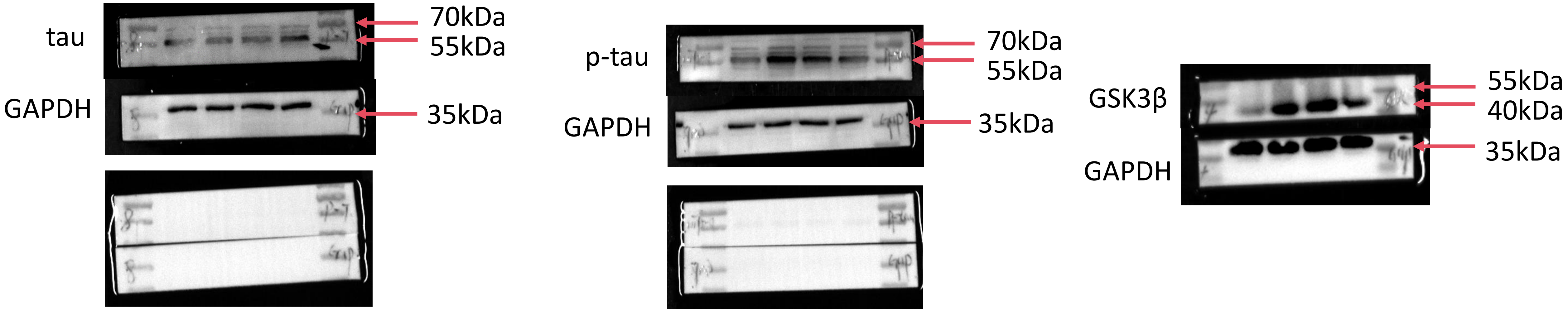

Figure. 5H (Hippocampus)

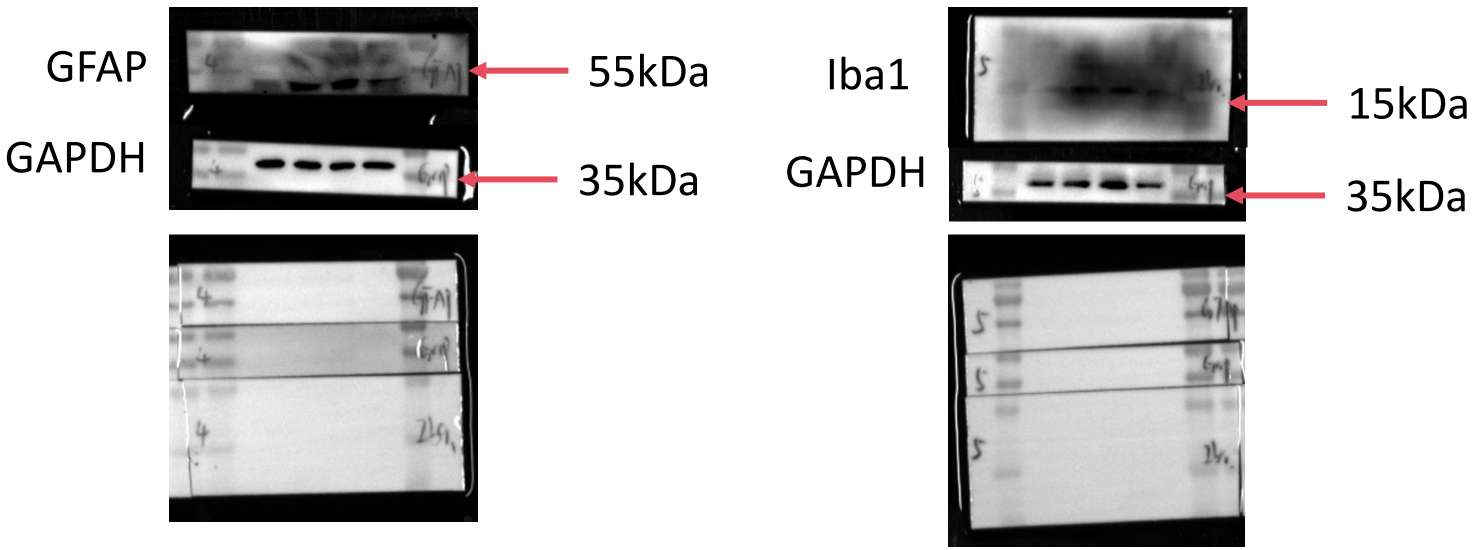

Figure. 5H (Cortex)

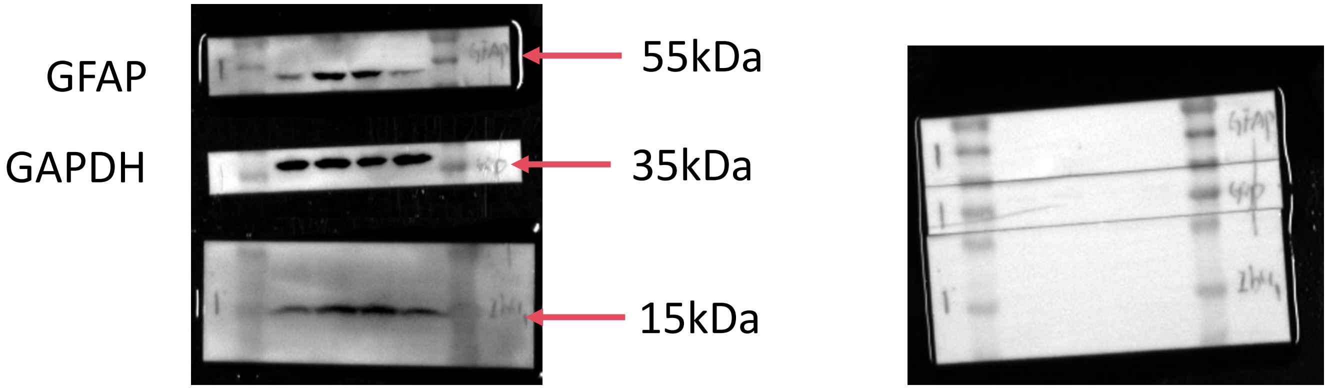

Figure. 6D (Hippocampus)

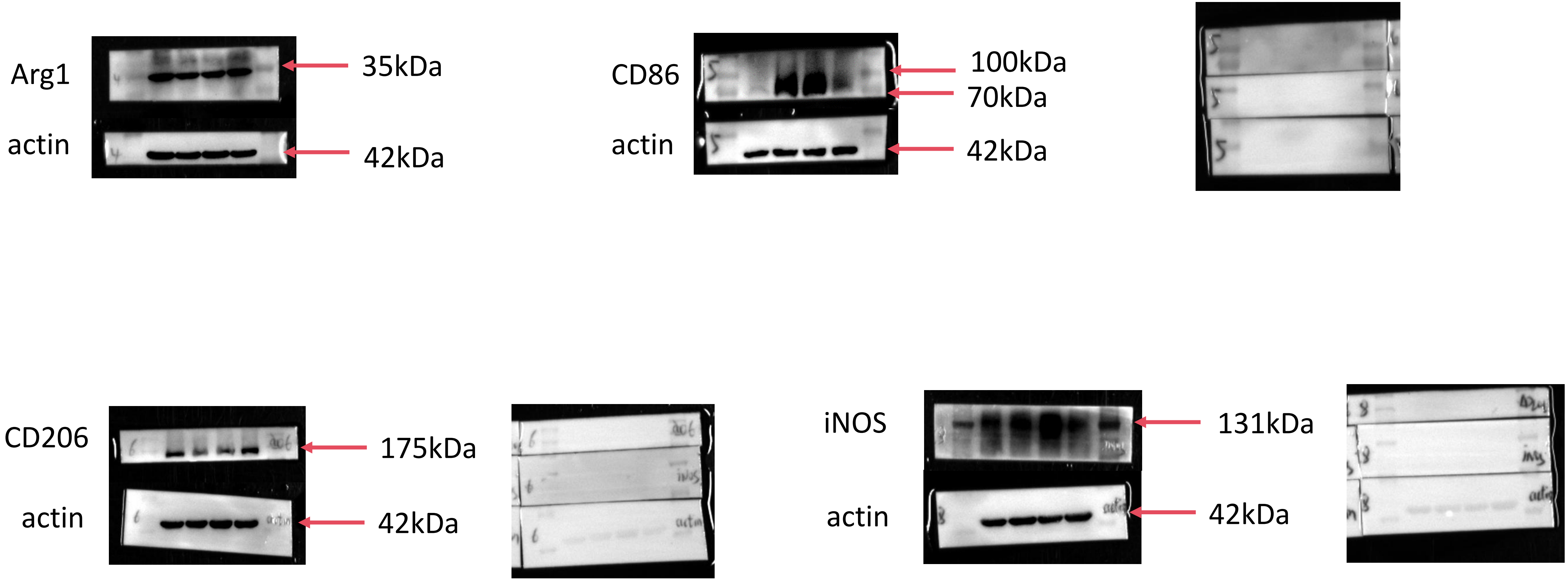

Figure. 6I (Cortex)

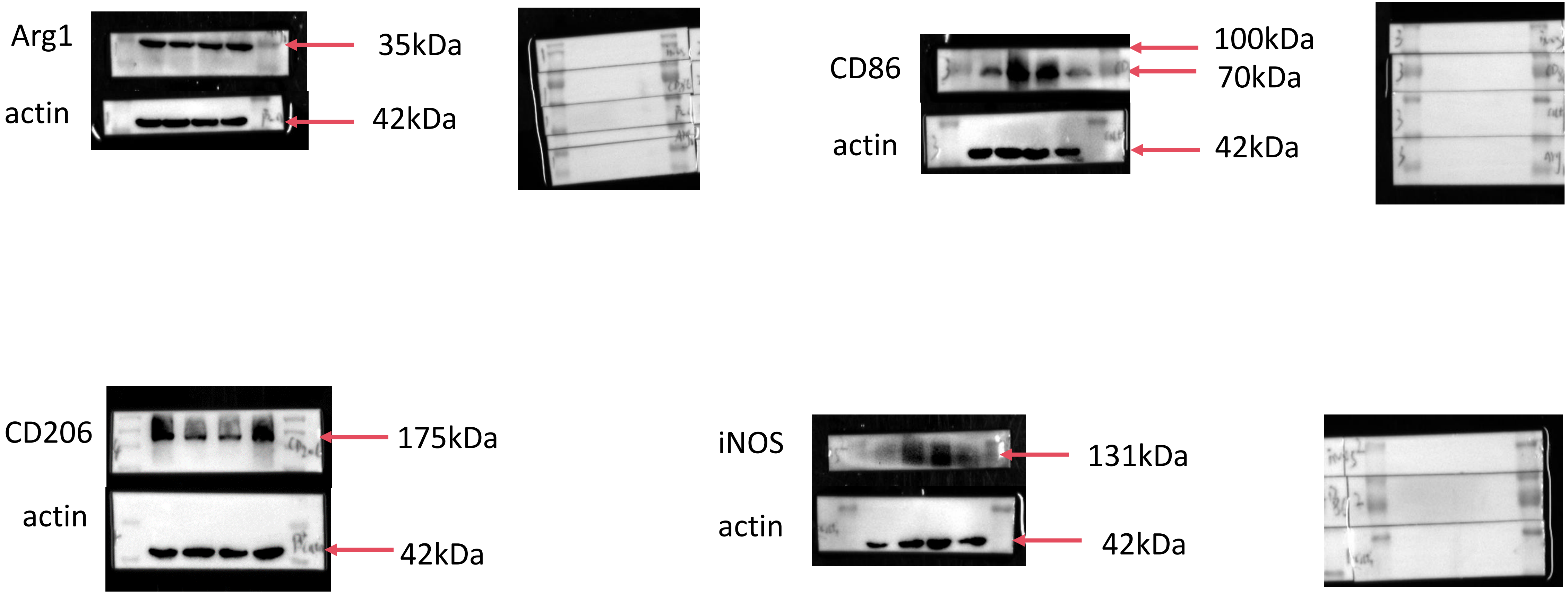

Figure. 7E (Hippocampus)

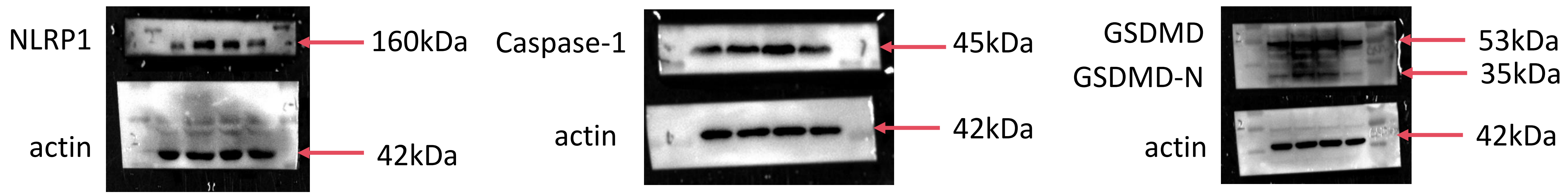

Figure. 7J (Cortex)

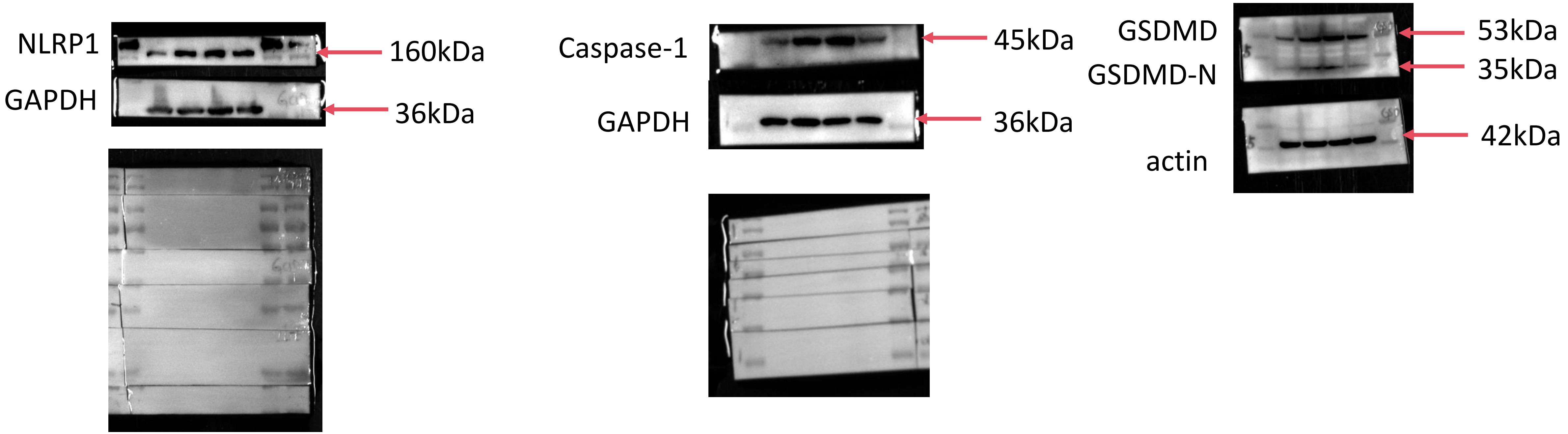

Figure. S5A

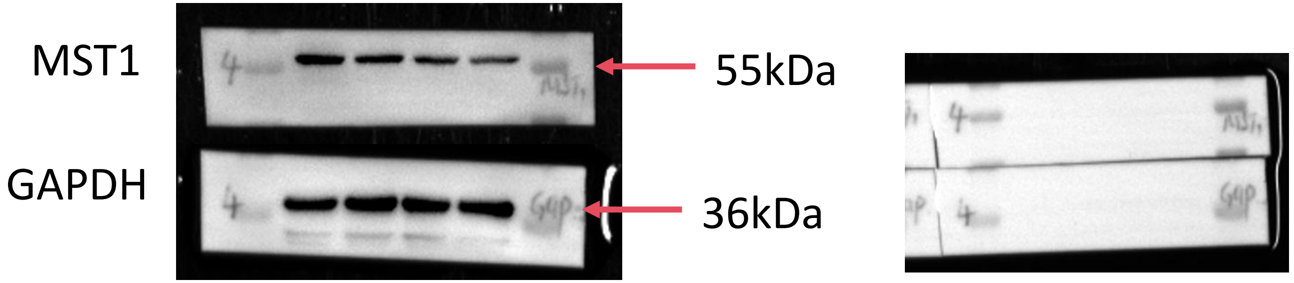

Figure. 7O

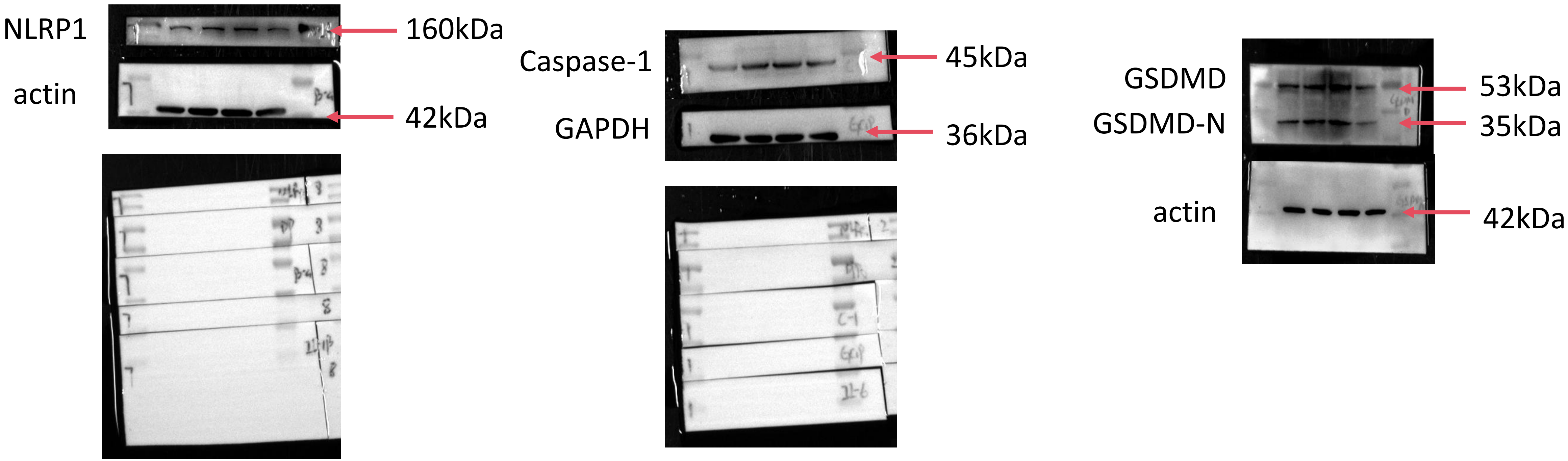

Figure. 8E

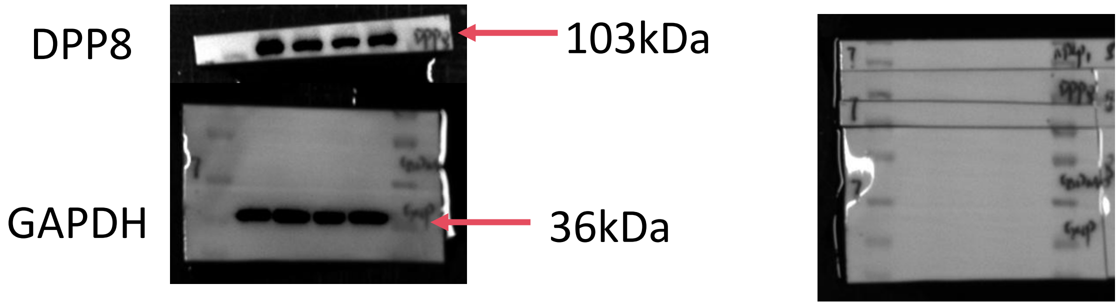

Figure. 8G

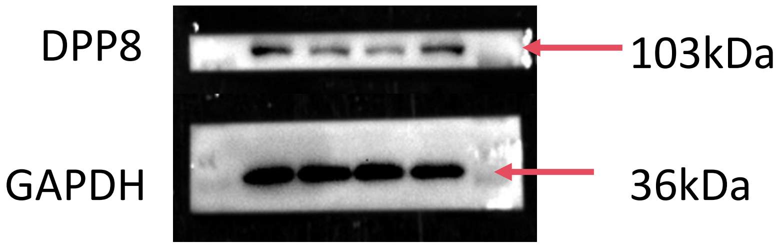

Figure. S8A

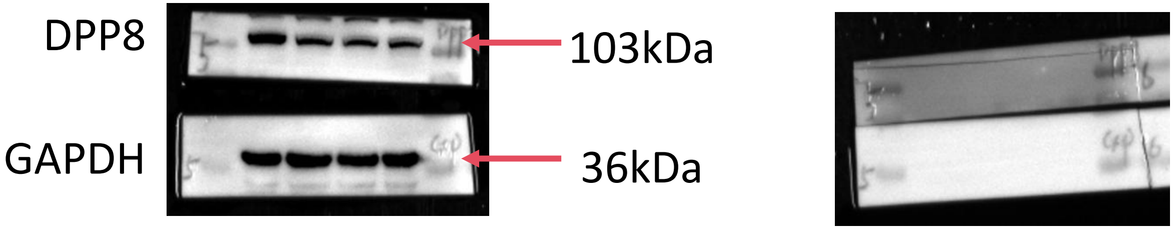

Figure. 9B

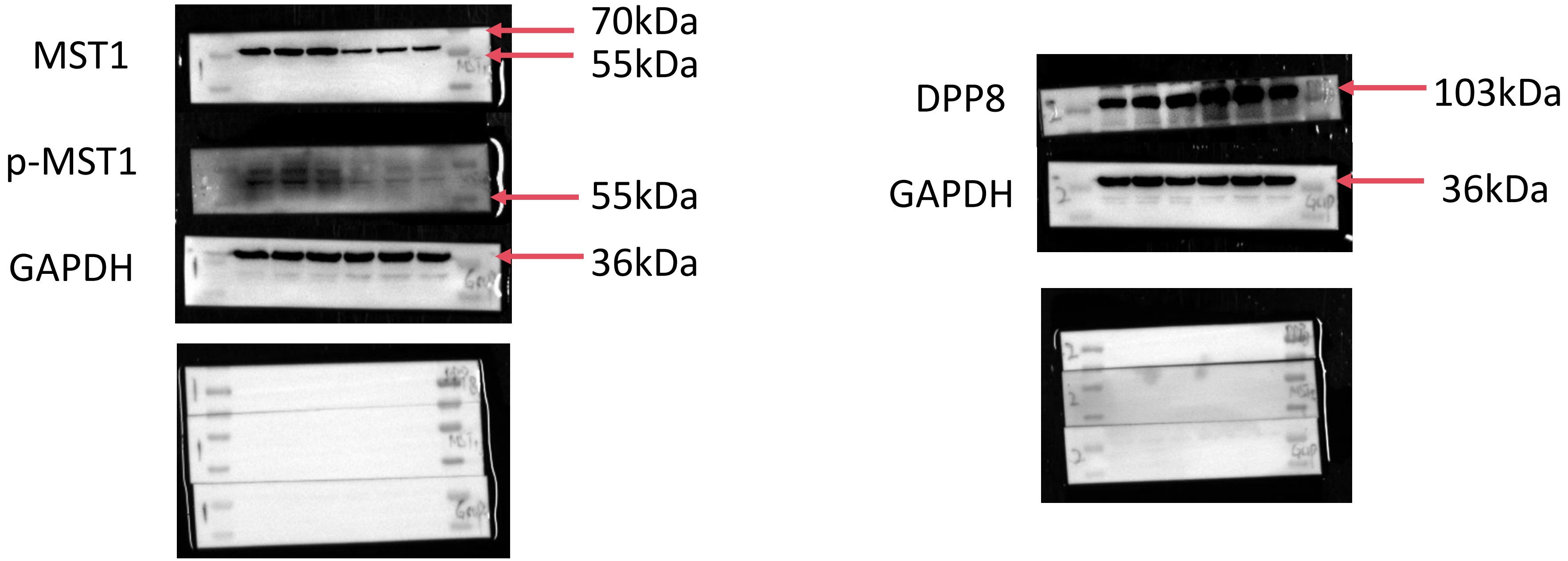

Figure. 9H

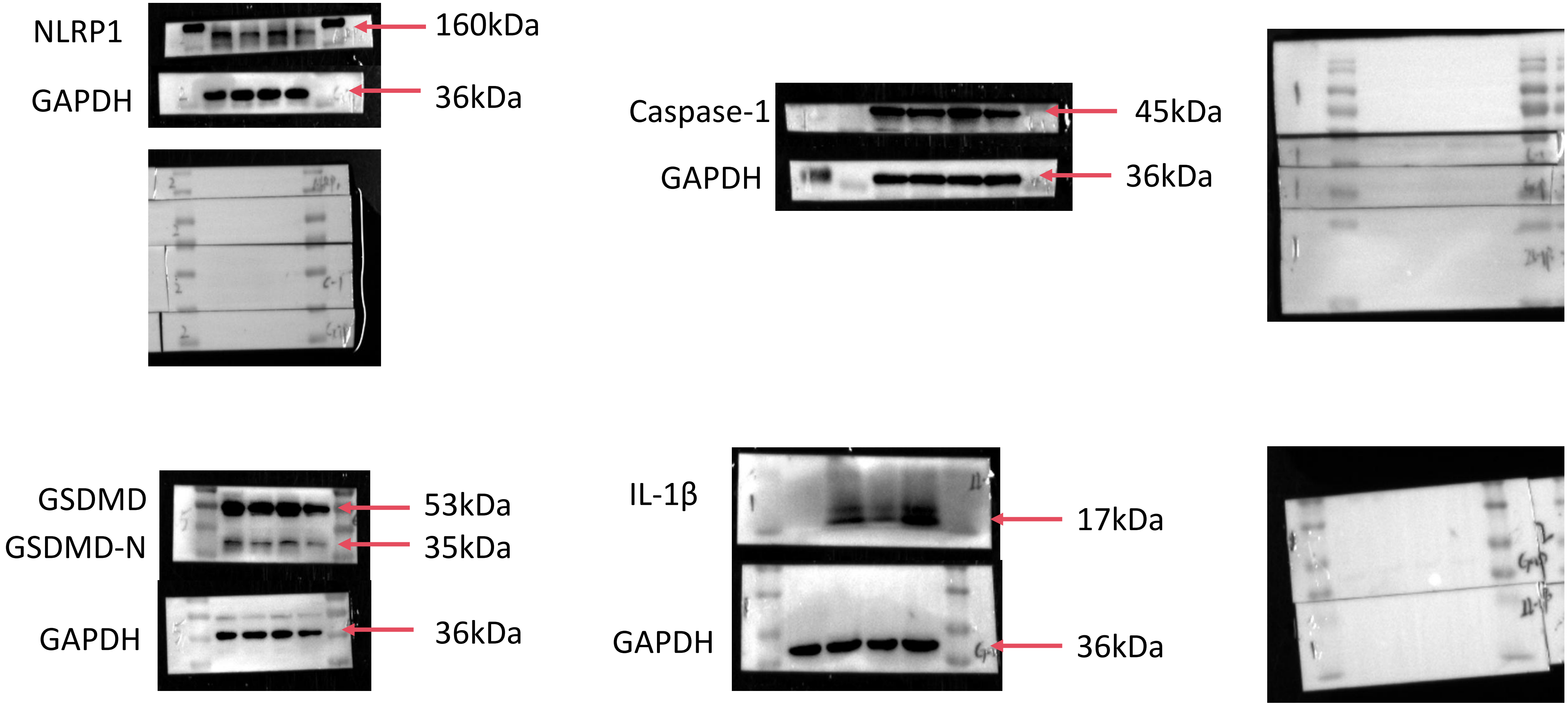
NLRP1
160kDa
GAPDH
36kDa
Caspase-1
45kDa
GAPDH
36kDa
GSDMD
53kDa
GSDMD-N
35kDa
GAPDH
36kDa
IL-1β
17kDa
GAPDH
36kDa
