# Supplementary material for: MST1 promotes microglial pyroptosis and neuroinflammation in alzheimer’s disease by regulating the novel DPP8/NLRP1/Caspase-1/GSDMD-N axis
Source: J Neuroinflammation. 2026 Feb 13;23:95. doi: 10.1186/s12974-026-03732-3 (PMC13005356; doi:10.1186/s12974-026-03732-3)
Supplement: Supplementary file 2 — Supplementary Material 2. [file 12974_2026_3732_MOESM2_ESM.docx]

**Supplementary methods:**

**1. Enzyme-linked immunosorbent assay (ELISA)**

Peripheral blood samples from humans and mice were centrifuged at 3500 rpm at 4°C for 15 min. The upper serum layer was carefully separated, and the levels of p-MST1 in the serum were measured using human-specific ELISA kits (Research Cloud Biotechnology, Jinan, China, KYY-64512H1, detection sensitivity:10pg/ml-450pg/ml ) and mouse-specific ELISA kits (Research Cloud Biotechnology, Jinan, China, KYY-4872M1, detection sensitivity: 5pg/ml-350pg/ml), respectively, according to the manufacturers’ instructions. Additionally, the level of p-Tau181 in human serum was detected using a human p-Tau181 ELISA kit (JONLNBIO, Shanghai, JL49331, detection sensitivity: 1.56-100pg/ml). Absorbance was determined at 450 nm using a microplate absorbance reader (Biotek, Vermont, USA).

After 24 h of co-culturing BV2 and HT22 cells, the cell culture medium was collected and centrifuged at 1000 rpm at 4°C for 5 min. The concentrations of IL-6 (Research Cloud Biotechnology, Jinan, China, KYY-0163M2, detection sensitivity: 3pg/ml-120pg/ml), IL-1β (Research Cloud Biotechnology, Jinan, China, KYY-0040M2, detection sensitivity: 31.25pg/ml-2000pg/ml), and TNF-α (Research Cloud Biotechnology, Jinan, China, KYY-0132M2, detection sensitivity: 25ng/L-800ng/L) in the cell culture supernatant were measured using an ELISA kit according to the manufacturer's instructions. Absorbance was determined at 450 nm using a microplate absorbance reader (Biotek, Vermont, USA). All ELISA measurements were performed with at least three technical replicates.

**2. Immunofluorescence staining and image analysis of brain tissue sections.**

In this study, all brain tissue sections were processed using strictly standardized staining protocols across different staining procedures to minimize technical variability. Image acquisition and quantitative analysis were performed in a blinded manner, with sample group information concealed to reduce subjective bias. For samples processed within the same batch, identical excitation light intensity, exposure time, and magnification were maintained, and all images were saved in TIFF format. Each experimental group included at least three biological replicates, with 2–4 brain sections obtained from each mouse. For each section, 2–4 non-overlapping fields of view were randomly selected within the regions of interest, with particular focus on the hippocampal CA1, CA3, DG, and cortical regions, to ensure the representativeness and reliability of the data.

**3. Proteomic analysis process and parameters based on TMT**

The proteomics data in this study were subjected to systematic quality control and analysis. Raw mass spectrometry data (*.raw) were acquired using an EASY-nLCTM–HFX liquid chromatography–tandem mass spectrometry system and directly imported into Proteome Discoverer version 2.5 for database searching, peptide identification, and protein quantification. The Mus_musculus_uniprot_2023_3_13. fasta database was used, and protein quantification was performed using the TMT reporter ion–based quantification method. Database search parameters were set as follows: trypsin was specified as the proteolytic enzyme, with up to two missed cleavage sites allowed; the mass tolerances for precursor and fragment ions were set to 10 ppm and 0.02 Da, respectively. Carbamidomethylation of cysteine residues (+57.021 Da) was defined as a fixed modification, whereas oxidation of methionine (+15.995 Da), TMT labeling of lysine residues (+304.207 Da), as well as protein N-terminal acetylation, N-terminal TMT labeling, methionine excision, and methionine excision followed by acetylation were set as variable modifications. To ensure high confidence in protein identification, search results were filtered at both the peptide and protein levels using a 1% false discovery rate (FDR) and a 99% confidence threshold, and only proteins identified with at least one unique peptide were retained. Differentially expressed proteins were identified using Student’s *t*-test, with significance defined as *p* < 0.05 and log_2_(fold change) ≥ 0.26 or ≤ −0.26. Finally, protein functional annotation and pathway analysis were performed using InterProScan, COG, and KEGG, and differentially expressed proteins were further analyzed by volcano plot visualization, hierarchical clustering, and GO/KEGG enrichment analyses.

**Supplementary figures:**

**Supplementary figure 1**


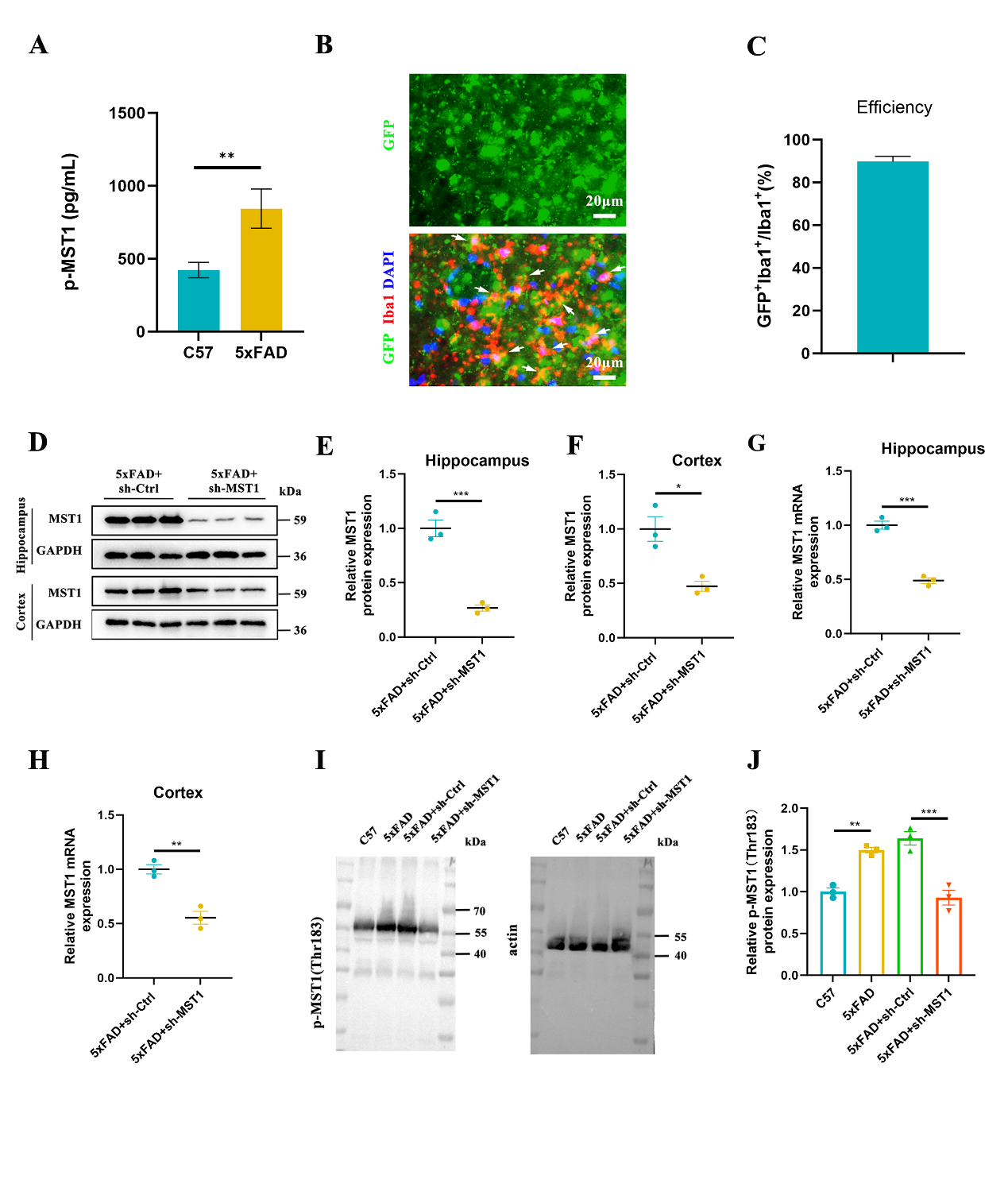


**Figure S1. The knockdown level of MST1 in hippocampal and cortical tissues was verified one month after AAVs injection.**

**A.** Quantitative analysis of p-MST1 levels in the cerebrospinal fluid of C57 and 5xFAD mice (n = 3 mice per group). **B.** Representative confocal images showing marker expression for the indicated AAVs (scale bar is 20µm). **C.** Quantifications showing microglia transduction efficiency (n = 3 mice per group)**. D-F.** Representative Western blot images illustrating relative protein levels of MST1 in the hippocampus and cerebral cortex after AAVs injection (n = 3 mice per group). **G.** RT-qPCR revealed the expression levels of MST1 mRNA in hippocampal tissues after AAVs injection (n = 3 mice per group). **H.** RT-qPCR revealed the expression levels of MST1 mRNA in cortical tissues after AAVs injection (n = 3 mice per group). **I-J.** Representative immunoblot images showing p-MST1 (Thr183) expression and corresponding quantitative analysis across experimental groups (n = 3 mice per group). Two-tailed unpaired Student's t-tests and One-way ANOVA with Tukey's multiple comparison test were used to evaluate statistical significance. Data are expressed as mean ± SEM, ***p* < 0.01, ****p* < 0.001.

**Supplementary figure 2**


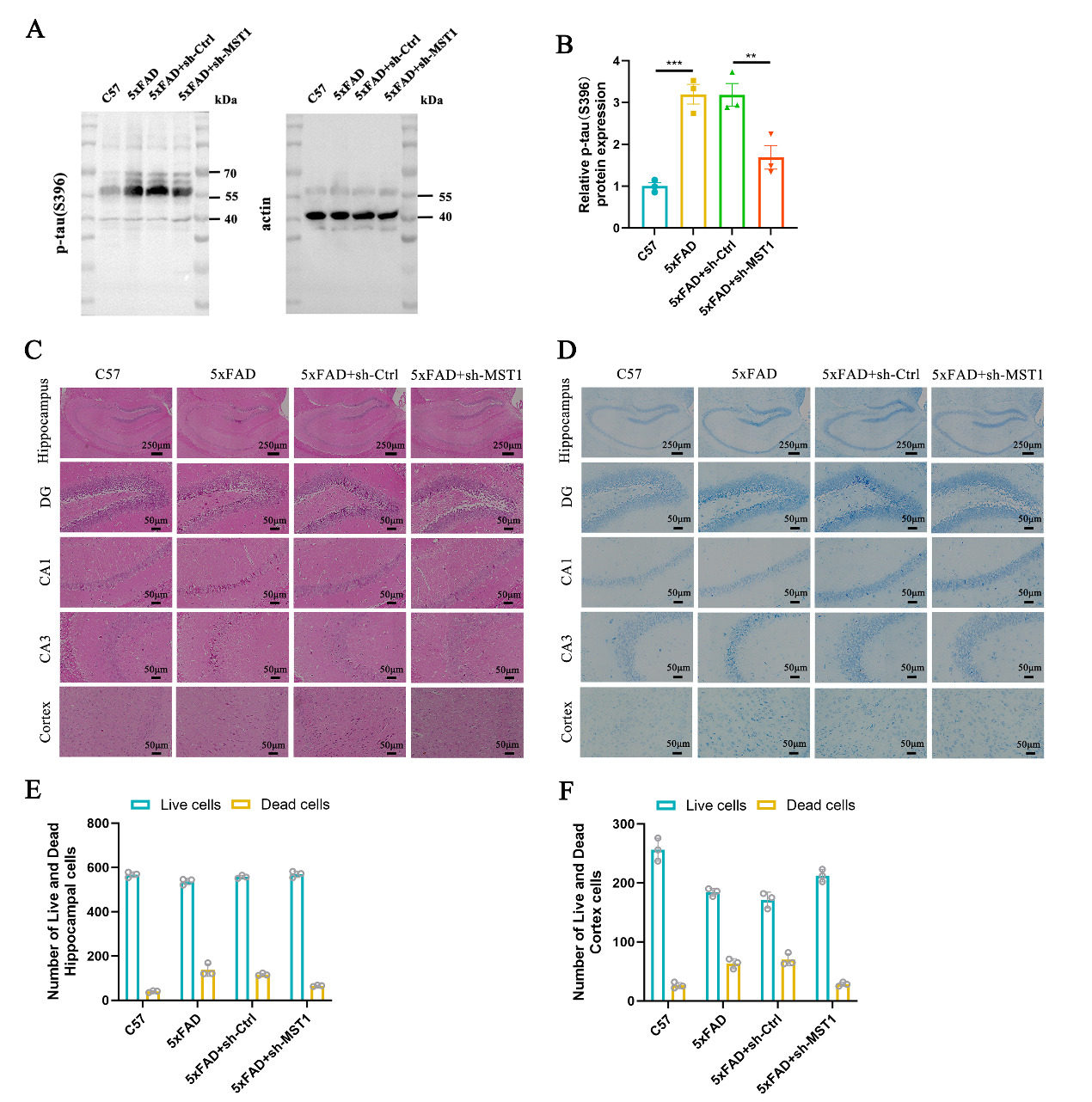


**Figure S2. Knockdown of MST1 reduces neurodegeneration in 5xFAD mice.**

**A-B.** Representative immunoblot images showing p-tau (S396) expression and corresponding quantitative analysis across experimental groups (n = 3 mice per group). **C.** Representative images of HE staining in the hippocampus (DG, CA1, and CA3 subregions) and cortical areas (n = 3 mice per group, original magnification: × 10, local magnification: × 40, Magnified multiple scale bar is 250 µm and enlarged images scale bar is 50 µm).  **D.** Representative images of Nissl staining in the hippocampus (DG, CA1, and CA3 subregions) and cortical areas (n = 3 mice per group, original magnification: × 10, local magnification: × 40, scale bar is 250 µm and enlarged images scale bar is 50 µm). **I-J.** Absolute numbers of live and dead cells in the hippocampal and cortical regions of the mouse brain across different experimental groups.

**Supplementary figure 3**

**
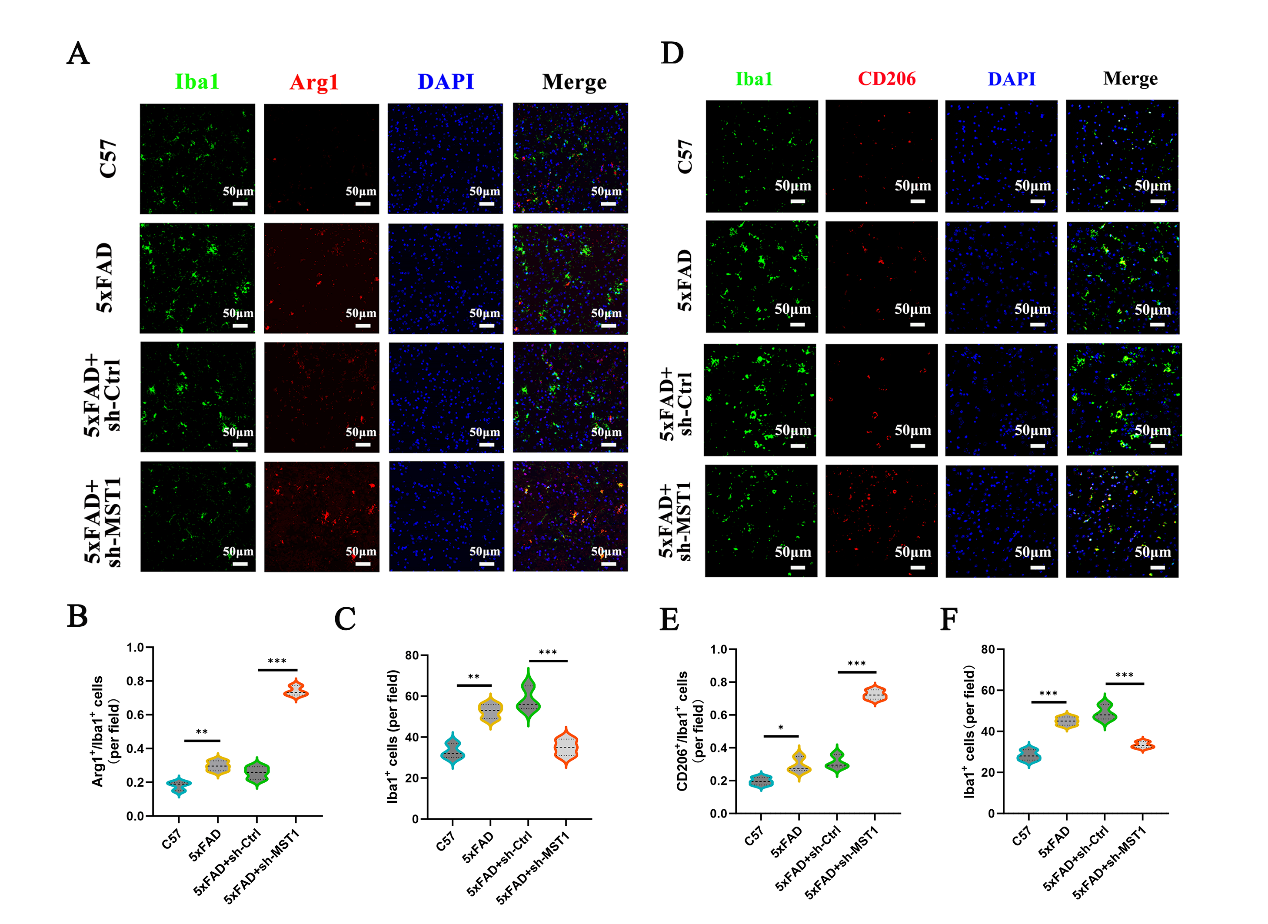
**

**Figure S3. MST1 knockdown reduces the number of iNOS-positive microglia, thereby suppressing microglial activation.**

**A.** Representative images of double immunofluorescence staining for Iba1 (green) and Arg1 (red) in the cortex, with DAPI (blue) used for nuclear counterstaining (n = 3 mice; original magnification: × 40, scale bar is 50μm). **B.** The number of Arg1-positive cells/the number of Iba1-positive cells per field (n = 3 mice per group; 2 sections/mouse; 3 fields/section; data averaged per mouse). **C.** The number of Iba1-positive cells per field (n = 3 mice per group; 2 sections/mouse; 3 fields/section; data averaged per mouse). **D.** Representative images of double immunofluorescence staining for Iba1 (green) and CD206 (red) in the cortex, with DAPI (blue) used for nuclear counterstaining (n = 3 mice; original magnification: × 40, scale bar is 50μm). **E.** The number of CD206-positive cells/the number of Iba1-positive cells per field (n = 3 mice per group; 2 sections/mouse; 3 fields/section; data averaged per mouse). **F.** The number of Iba1-positive cells per field (n = 3 mice per group; 2 sections/mouse; 3 fields/section; data averaged per mouse). One-way ANOVA with Tukey's multiple comparison test was employed. Data are expressed as mean ± SEM, **p* < 0.05, ***p* < 0.01, ****p* < 0.001.

**Supplementary figure 4**


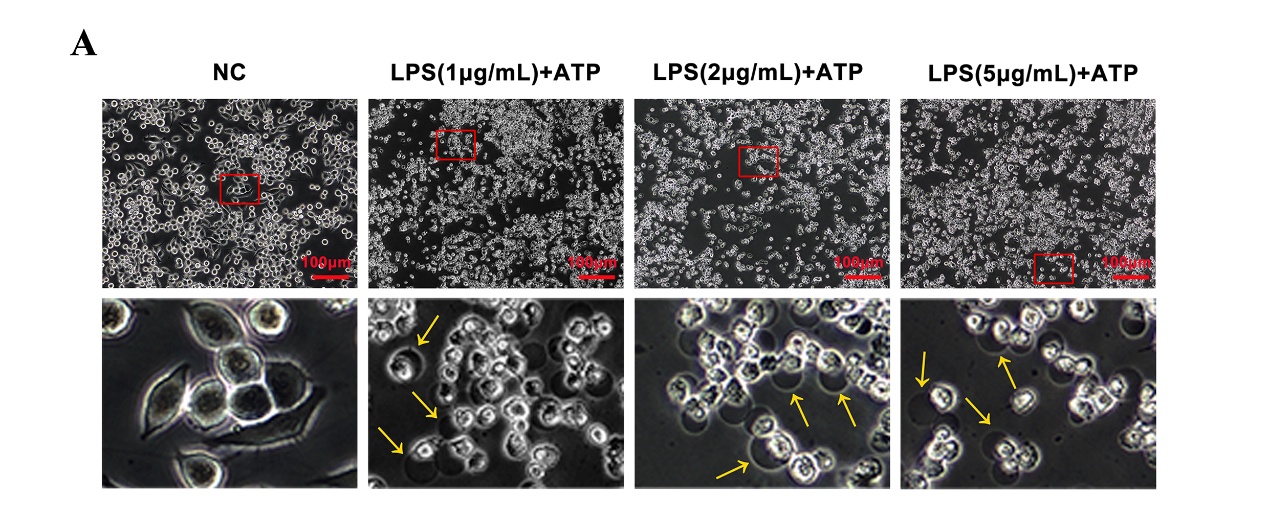


**Figure S4. LPS and ATP induced pyroptosis in BV2 cells.**

BV2 cells were treated with LPS at different concentrations (1 μg/mL, 2 μg/mL, 3 μg/mL) for 23 hours, followed by treatment with ATP (5 mM) for 1 hour, and then changes in cell morphology were observed. **A.** Microscopic images showing pyroptosis in BV2 cells after LPS and ATP treatment (scale bar is 100μm). Yellow arrows point to pyroptotic cells, which are characterized by the presence of pyroptotic blebs, cytoplasmic swelling, and membrane rupture.

**Supplementary figure 5**


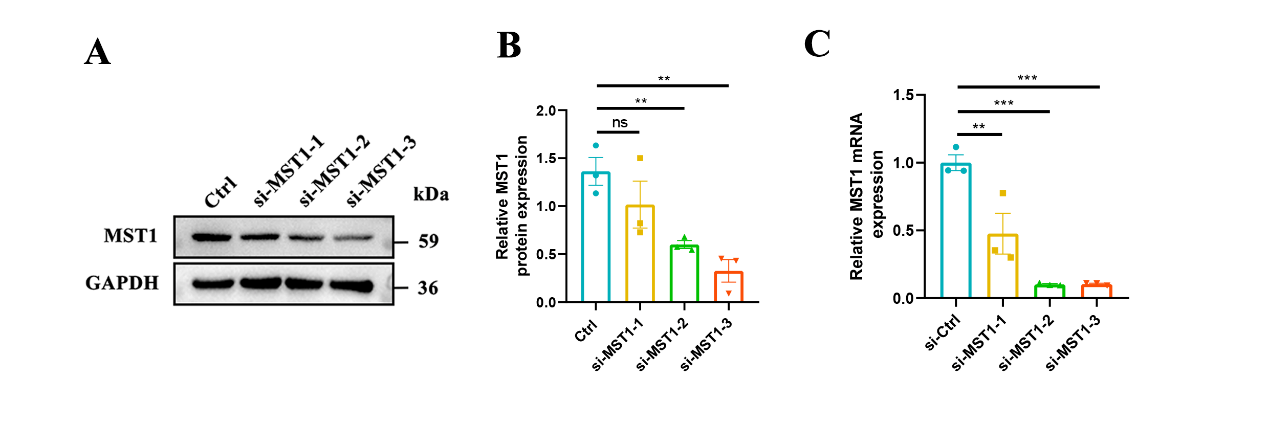


**Figure S5. Verification of transfection efficiency of siMST1 in BV2 cells.**

**A–B.** Representative Western blot images showing relative protein levels of MST1 in BV2 cells (n = 3 per group). **C.** RT-qPCR revealed the expression levels of MST1 mRNA in BV2 cells (n = 3 per group). Two-tailed unpaired Student's t-tests were used to evaluate statistical significance. Data are expressed as mean ± SEM, ***p* < 0.01, ****p* < 0.001, *ns*, no significance.

**Supplementary figure 6**


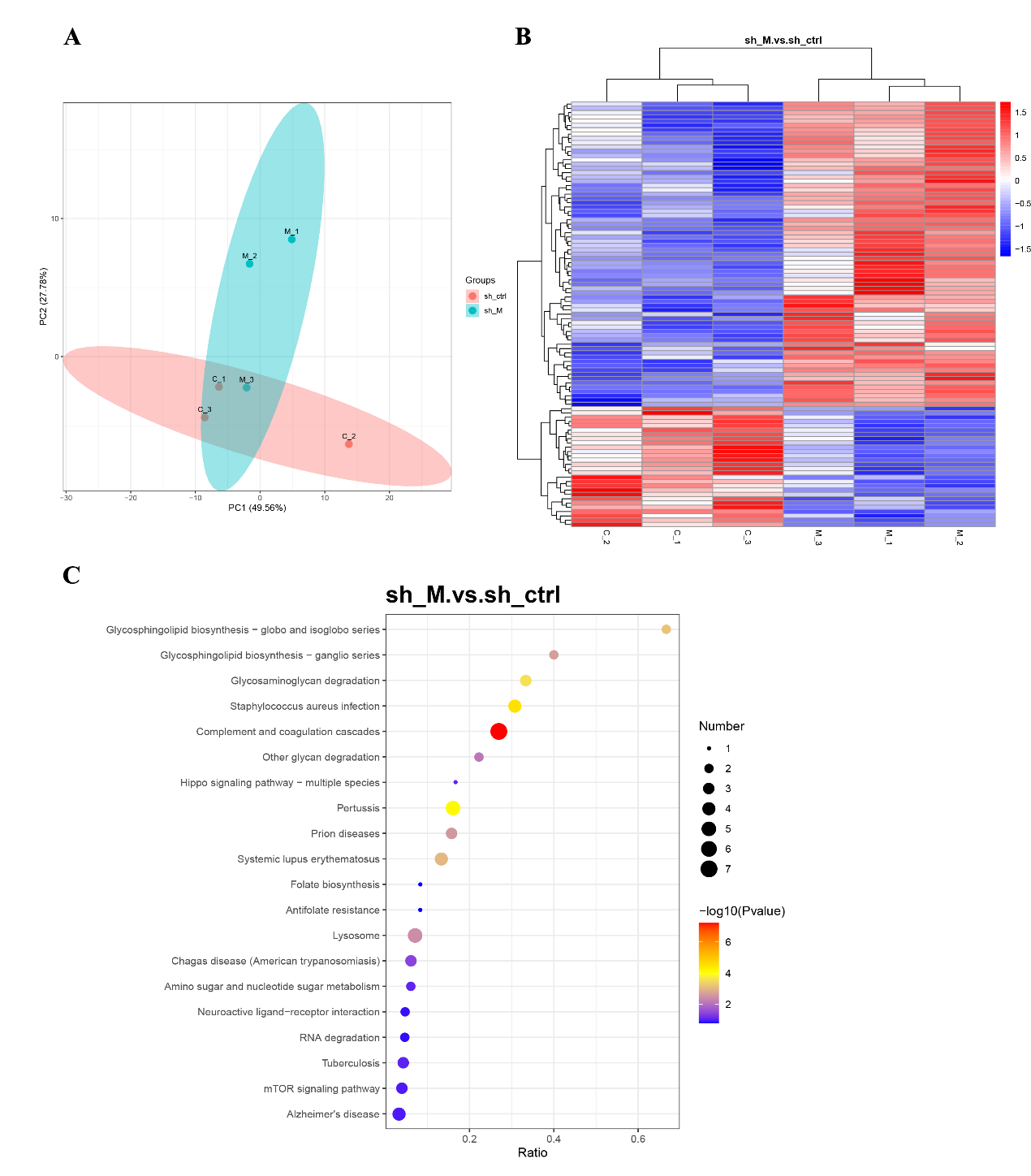


**Figure S6. TMT proteomic analysis of hippocampal tissue in 5xFAD mice after MST1 knockdown**

**A.** Principal component analysis (PCA) was performed to provide an overall assessment of global proteomic differences among experimental groups and the degree of variability within each group. The x-axis (PC1) and y-axis (PC2) represent the scores of the first and second principal components, respectively, while the colors of the data points indicate different experimental groups. Note: C1, C2, and C3 represent samples from the 5xFAD + sh-Ctrl group, while M1, M2, and M3 represent samples from the 5xFAD + sh-MST1 group. **B.** The clustered heatmap of differentially expressed proteins (DEPs) shows the cluster analysis of the relative content of differentially expressed proteins in each sample, revealing the up - regulation and down - regulation of proteins in different samples. The vertical direction represents the clustering of samples, and the horizontal direction represents the clustering of proteins. The shorter the clustering branch, the higher the similarity. **C.** The bubble plot of DEPs shows the significantly enriched pathways in KEGG analysis.

**Supplementary figure 7**


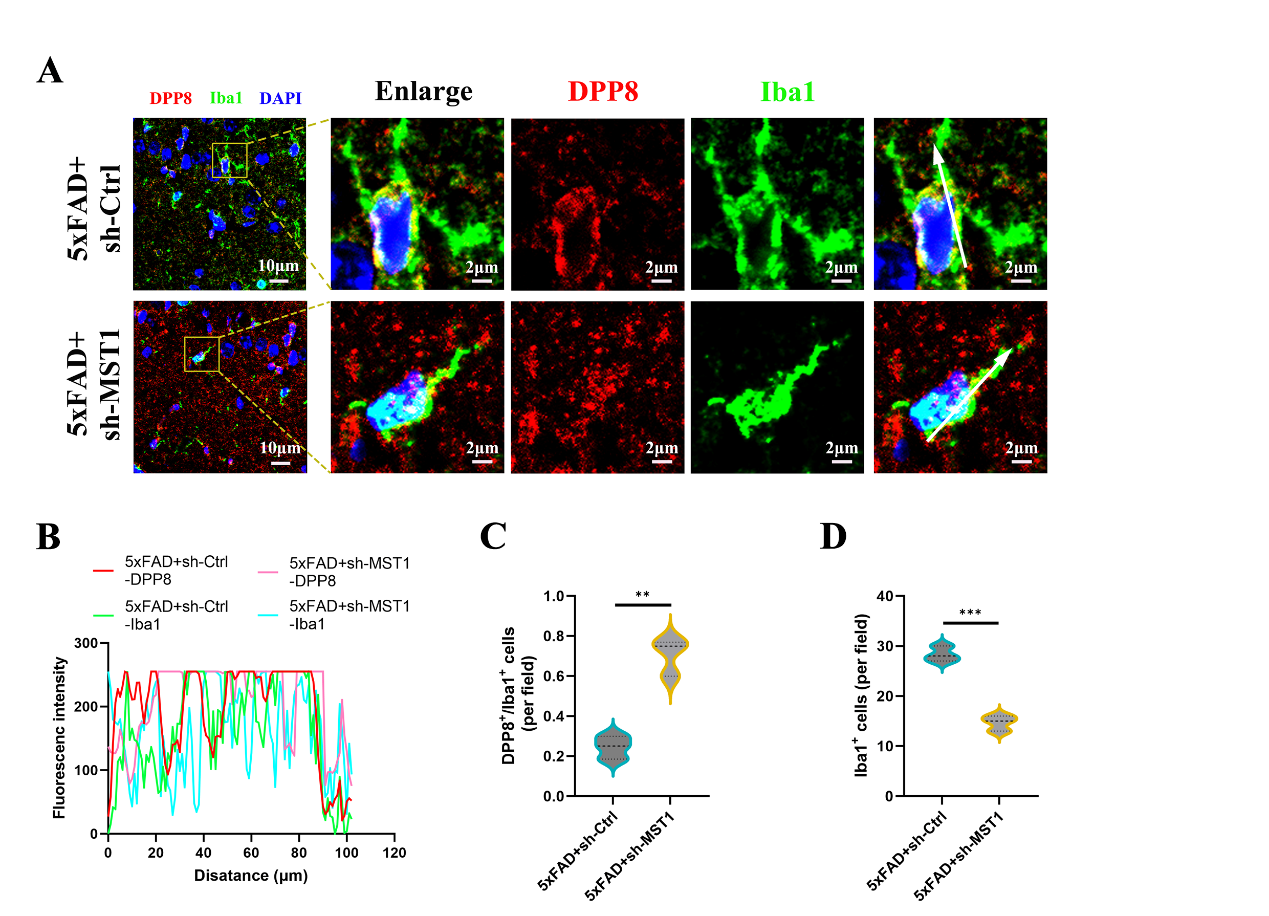


**Figure S7.** **MST1 knockdown promotes DPP8 expression in microglia.**

**A.** Representative images of double immunofluorescence staining of Iba1 (red) and iNOS (green) in the cortex. DAPI (blue) was used for nuclear counterstaining (n = 3 mice; Images were acquired using a confocal laser scanning microscope equipped with a 100× oil-immersion objective; scale bar is 10μm; scale bar for enlarged insets is 2μm). B. Fluorescence intensity of Iba1 and iNOS in cells indicated by the white arrows. **C.** The number of DPP8-positive cells/the number of iba1-positive cells per field (n = 3 mice per group; 1 sections/mouse; 3 fields/section; data averaged per mouse). **D.** The number of iba1-positive cells per field (n = 3 mice per group; 1 sections/mouse; 3 fields/section; data averaged per mouse). Two-tailed unpaired Student's t-tests were used to evaluate statistical significance. Data are expressed as mean ± SEM, ***p* < 0.01, ****p* < 0.001.

**Supplementary figure 8**


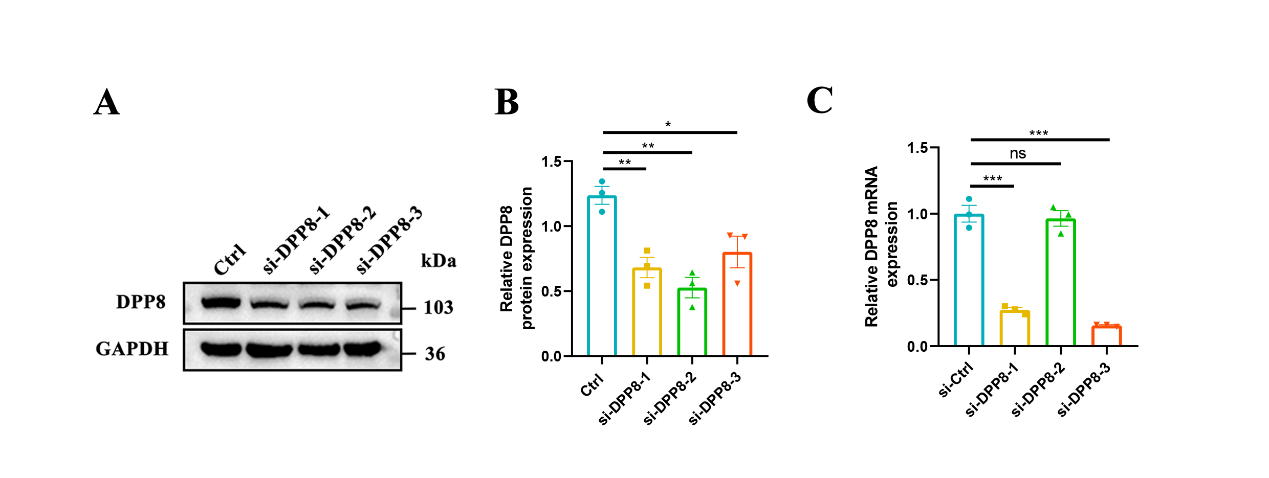


**Figure S8. Verification of transfection efficiency of siDPP8 in BV2 cells.**

**A–B.** Representative Western blot images showing relative protein levels of DPP8 in BV2 cells (n = 3 per group). **C.** RT-qPCR revealed the expression levels of DPP8 mRNA in BV2 cells (n = 3 per group). Two-tailed unpaired Student's t-tests were used to evaluate statistical significance. Data are expressed as mean ± SEM, ***p* < 0.01, ****p* < 0.001, *ns*, no significance.
